# Retrograde BDNF to TrkB signaling promotes synapse elimination in the developing cerebellum

**DOI:** 10.1038/s41467-017-00260-w

**Published:** 2017-08-04

**Authors:** Myeongjeong Choo, Taisuke Miyazaki, Maya Yamazaki, Meiko Kawamura, Takanobu Nakazawa, Jianling Zhang, Asami Tanimura, Naofumi Uesaka, Masahiko Watanabe, Kenji Sakimura, Masanobu Kano

**Affiliations:** 1 Department of Neurophysiology, Graduate School of Medicine, The University of Tokyo, Tokyo, 113-0033 Japan; 2 Department of Anatomy, Hokkaido University Graduate School of Medicine, Sapporo, 060-8638 Japan; 3 Department of Cellular Neurobiology, Brain Research Institute, Niigata University, Niigata, 951-8585 Japan

## Abstract

Elimination of early-formed redundant synapses during postnatal development is essential for functional neural circuit formation. Purkinje cells (PCs) in the neonatal cerebellum are innervated by multiple climbing fibers (CFs). A single CF is strengthened whereas the other CFs are eliminated in each PC dependent on postsynaptic activity in PC, but the underlying mechanisms are largely unknown. Here, we report that brain-derived neurotrophic factor (BDNF) from PC facilitates CF synapse elimination. By PC-specific deletion of BDNF combined with knockdown of BDNF receptors in CF, we show that BDNF acts retrogradely on TrkB in CFs, and facilitates elimination of CF synapses from PC somata during the third postnatal week. We also show that BDNF shares signaling pathway with metabotropic glutamate receptor 1, a key molecule that triggers a canonical pathway for CF synapse elimination. These results indicate that unlike other synapses, BDNF mediates punishment signal for synapse elimination in the developing cerebellum.

## Abstract (web summary)

During development, synapses are selectively strengthened or eliminated by activity-dependent competition. Here, the authors show that BDNF-TrkB retrograde signaling is a “punishment” signal that leads to elimination of climbing fiber-onto-Purkinje cell synapses in the developing cerebellum.

## Introduction

Precise formation of neural circuits during development is a basis for proper functions of the nervous system. Neuronal connections are diverse and redundant around birth, but they are refined during postnatal development by selective strengthening of necessary synapses and elimination of unnecessary redundant connections in activity-dependent manners^1–3^. This process, known as synapse elimination, is widely thought to be crucial for shaping immature neural circuits into functionally mature versions. Excitatory synapses between climbing fibers (CFs) and Purkinje cells (PCs) in the cerebellum have been a representative model of developmental synapse elimination in the mammalian nervous system^4–8^. CFs originate from neurons in the inferior olive of the medulla and make strong excitatory synapses onto proximal dendrites of PCs in the adult cerebellum^9, 10^. In the cerebellum of adult mice, most PCs are innervated by single CFs^9, 10^. However, in the cerebellum of neonatal mice, multiple CFs with similar strengths innervate the soma of each PC. The adult-type CF mono innervation is established through the processes in the four distinct phases during postnatal development. First, a single CF is selectively strengthened in each PC from postnatal day 3 (P3) to P7 (phase of functional differentiation)^11^. Second, only the strengthened CF, which is the “winner” of the homosynaptic competition during functional differentiation, undergoes translocation to growing PC dendrite from around P9 (phase of CF translocation)^12^. Third and fourth, in parallel with CF translocation, synapses of the other weaker CFs, the “losers” of the homosynaptic competition during functional differentiation, are eliminated from the PC soma in two distinct phases: the early phase from P7 to around P11 which proceeds independently of synaptogenesis from parallel fibers (PFs), the other excitatory input to PCs (early phase of CF elimination), and the late phase from around P12 to around P17 which requires normal formation of PF-PC synapses (late phase of CF elimination)^7, 8, 13^. Finally, after establishment of mono innervation of each PC by a “winner” CF, PF synapses are eliminated from proximal PC dendrites during P15 to around P30 and CF innervation further expands along PC dendrites^14^.

Several molecules have been identified to be involved in CF synapse elimination, including the type 1 metabotropic glutamate receptor (mGlu1)^15, 16^, Gαq^17^, phospholipase Cβ4 (PLCβ4)^18^, protein kinase Cγ (PKCγ)^19^, glutamate receptor δ2 (GluD2)^20, 21^, P/Q-type voltage-dependent Ca^2+^ channel (P/Q-VDCC)^22, 23^, NMDA receptor^24, 25^, and Arc/Arg3.1^26^. However, how signals from these molecules transferred to CFs and lead to elimination of redundant CF synapses remains largely unknown. Since key molecules such as mGlu1, Gαq, PLCβ4, PKCγ, and P/Q-VDCC function in postsynaptic PCs, some retrograde signals from PCs are thought to mediate their effects on CFs destined to be eliminated. Our recent work demonstrates that retrograde semaphorin signaling regulates synapse elimination in the cerebellum^27^. However, deletion of the semaphorin signaling does not block all the components of CF synapse elimination^27^ suggesting the involvement of other retrograde signaling molecules in CF synapse elimination.

As a candidate of such molecules, we focused on brain-derived neurotrophic factor (BDNF). BDNF plays crucial roles in many processes of brain development including neuronal survival, neurite outgrowth, and synaptic plasticity^28, 29^. BDNF binds to the B-type tyrosine kinase receptor (TrkB) and the p75 low affinity neurotrophin receptor (p75^NTR^). BDNF can modulate synaptic transmission not only anterogradely but also retrogradely^30^. Importantly, BDNF has been shown to be involved in synapse elimination at the neuromuscular junction^31^. Moreover, in TrkB knockout mice, PCs are reported to be innervated by multiple CFs at P14^32^ and at P20–P24^32, 33^, indicating that BDNF signaling is involved in CF synapse elimination in the cerebellum. However, it is not known how BDNF to TrkB signaling, which is widely known to support/strengthen synapses in many regions of the nervous system^29^, contributes to elimination of redundant CF synapses in the developing cerebellum. Moreover, it remains unclear from which cell-types BDNF is released and on which structure BDNF acts in the developing cerebellum.

To elucidate how BDNF signaling contributes to developmental CF synapse elimination, we generated conditional *Bdnf* knockout mice in which BDNF is deleted mainly in postsynaptic PCs (BDNF-PC-KO mice). We also performed lentivirus-mediated knockdown of BDNF specifically in PCs. To identify the receptor on which BDNF acts and the cell-type that expresses the receptor, we deleted either TrkB or p75^NTR^ in PCs or CFs. Our results show that BDNF derived from PCs retrogradely acts on TrkB on CFs and facilitate elimination of CF synapses from the PC soma after P16. Furthermore, we show that BDNF and mGlu1 function presumably along the same signaling pathway for CF synapse elimination. Thus, these results strongly suggest that retrograde BDNF to TrkB signaling regulated by mGlu1 is required for the late phase of CF synapse elimination in the developing cerebellum.

## Results

### Multiple CF innervation of PCs in mature BDNF-PC-KO mice

We first examined developmental profiles of the expression of BDNF and its putative receptors, TrkB, p75^NTR^, and SORT1, in the cerebellum. Mature BDNF is known to be produced by proteolysis of its precursor pro BDNF^34, 35^. BDNF has been reported to be expressed in PCs^36^ and granule cells^37^ of the cerebellum. Our western blot analyses of cerebellar tissues show that the expression of mature BDNF was very low at P7, significantly increased at P15, and then further increased at P28 (Supplementary Fig. 1a). In contrast, the expression of TrkB was constant from P7 to P28 (Supplementary Fig. 1b), whereas that of p75^NTR^ decreased from P7 to P15 and from P15 to P28 (Supplementary Fig. 1c). The expression of another putative BDNF receptor, SORT1^38^, increased only slightly from P7 to P15 (Supplementary Fig. 1d). These results show that BDNF and its receptors are abundantly expressed in the cerebellum at least after P15.

We generated PC-selective BDNF knockout mice (BDNF-PC-KO mice) (Supplementary Fig. 2a, b) and confirmed a significant reduction of mature BDNF protein in the cerebellum of BDNF-PC-KO mice (Supplementary Fig. 2c) and the deletion of BDNF only in genomic DNA prepared from the cerebellum (Supplementary Fig. 2d) of BDNF-PC-KO mice. We found that foliation, thickness and morphology of the molecular layer (ML) and granule cell layer (GL) of the cerebellum (Supplementary Fig. 3a, c), morphology of the soma and dendrites of PCs (Supplementary Fig. 3e, g), and morphology of Bergmann glial fibers (Supplementary Fig. 3j, l) were normal in BDNF-PC KO mice. Furthermore, distribution of vesicular glutamate transporter 2 (VGluT2)-positive CF terminals (Supplementary Fig. 3b, d–h) and that of vesicular glutamate transporter 1 (VGluT1)-positive PF terminals (Supplementary Fig. 3i, k) were not altered in BDNF-PC-KO mice. Our quantitative measurements of the thickness and the area of the ML, GL, and white matter showed no significant difference between the genotypes (Supplementary Fig. 3m, n). Thus gross anatomy of the cerebellum, morphology of PCs and Bergmann glia, and distribution of CF and PF synaptic terminals appear normal in BDNF-PC-KO mice.

**Fig. 1:**
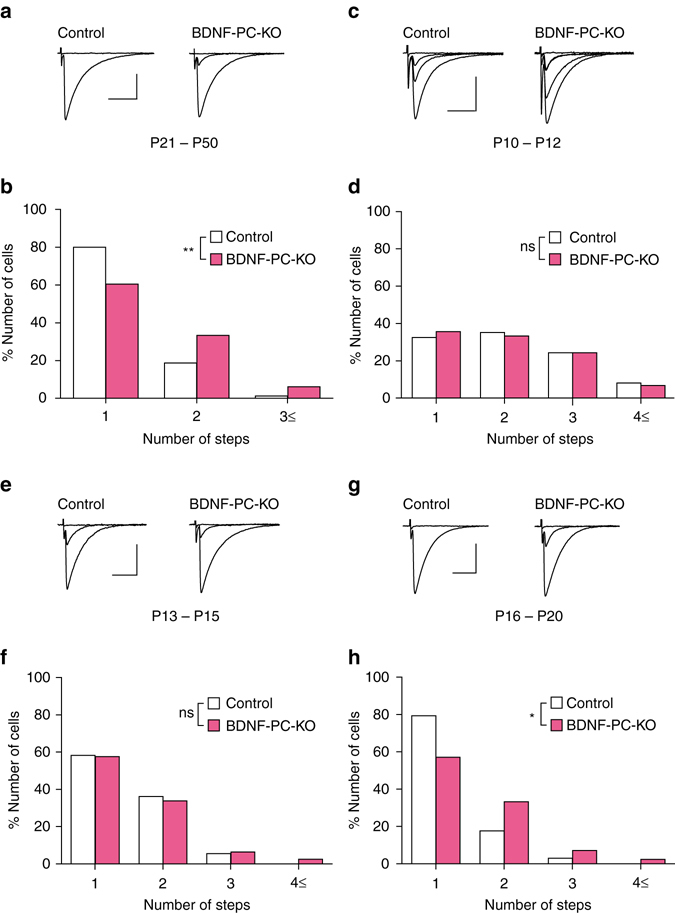
Impairment of CF synapse elimination in BDNF-PC-KO mice after P16. **a** Representative traces of CF-EPSCs recorded in a PC from a control (*left*, P21) and a BDNF-PC-KO (*right*, P21) mouse. Holding potential was −10 mV. Single traces recorded at different stimulus intensities were superimposed. Scale bars, 10 ms and 1 nA. **b** Frequency distribution histogram for the number of CFs innervating each PC in control (*open columns*, *n* = 80 PCs from 10 mice) and BDNF-PC-KO (*filled columns*, *n* = 81 PCs from 12 mice) mice during P21–P50 (*P* = 0.0055, Mann–Whitney *U*-test). **P < 0.01. **c**–**h** Representative traces of CF-EPSCs (**c**, **e**, **g**) and frequency distribution histograms for the number of CFs innervating each PC (**d**, **f**, **h**) for control (*open columns*) and BDNF-PC-KO (*filled columns*) PCs during P10–P12 (**c**, **d**), P13–P15 (**e**, **f**), and P16–P20 (**g**, **h**). CF-EPSCs were recorded in cerebellar slices from a mouse at P11 (**c**), P14 (**e**), or P17 (**g**). Holding potential was −10 mV. Single traces recorded at different stimulus intensities were superimposed. Scale bars, 10 ms and 1 nA. Sample sizes for frequency distribution histograms were 37 PCs from 5 control mice and 45 PCs from 5 BDNF-PC-KO mice for P10–P12 (*P* = 0.788, Mann–Whitney *U*-test) (**d**), 72 PCs from 6 control mice and 67 PCs from 6 BDNF-PC-KO mice for P13–P15 (*P* = 0.553, Mann–Whitney *U*-test) (**f**) and 34 PCs from 5 control mice and 42 PCs from 6 BDNF-PC-KO mice for P16–P20 (*P* = 0.037, Mann–Whitney *U*-test) (**h**). *P < 0.05, ns indicates no significant difference between the groups

We made whole-cell recordings from PCs, stimulated CFs, and recorded CF-mediated excitatory postsynaptic currents (EPSCs) in cerebellar slices prepared from young adult mice during P21–P50. We moved the stimulation electrode systematically around the PC soma under recording and increased the stimulus intensity gradually at each stimulation site. As exemplified in Supplementary Fig. 4, CF-EPSCs were elicited with discrete steps when the stimulus intensity was increased. We estimated the number of CFs innervating each PC from discrete CF-EPSCs elicited in each PC^19^. In wild-type littermates of BDNF-PC-KO mice (control mice), 80% of PCs were innervated by single CFs while only 20% were innervated by two or more CFs. In contrast, two or three steps of CF-EPSCs were found in 39.5% of PCs in BDNF-PC-KO mice (Fig. 1a, b). Frequency distribution histograms clearly show that the percentage of PCs innervated by multiple CFs was significantly higher in BDNF-PC-KO mice than in control mice (Fig. 1b).

We found that basic properties of CF-EPSCs were not affected in BDNF-PC-KO mice (Supplementary Table 1). We divided CFs into three groups, namely CFs for mono-innervated PCs (CF-mono), CF that generates the largest CF-EPSCs in each multiply innervated PC (CF-multi-S), and CFs that generate smaller CF-EPSC in each multiply innervated PC (CF-multi-W)^6, 11, 12^. There were no significant differences in the EPSC parameters for CF-mono, CF-multi-S, and CF-multi-W between control and BDNF-PC-KO mice (Supplementary Table 1). To test whether the selective strengthening of single CFs is affected in BDNF-PC-KO mice, we calculated the two parameters, disparity ratio and disparity index, for each multiply innervated PC^11^. The disparity index and ratio were not different between control and BDNF-PC-KO mice (Supplementary Table 2), indicating that the selective strengthening of single CFs was not affected by the lack of BDNF in PCs.

### BDNF contributes to CF synapse elimination after P16

To determine the developmental stage of CF synapse elimination to which BDNF contributes, we analyzed the number of CFs innervating each PC from P10 to P20. The number of CFs on each PC was not altered at P10–P12 (Fig. 1c, d) and at P13–P15 (Fig. 1e, f) but was significantly higher at P16–P20 (Fig. 1g, h) in BDNF-PC-KO mice when compared with control mice. We found no significant difference in basic EPSC parameters for CF-mono, CF-multi-S, and CF-multi-W between control and BDNF-PC-KO mice from P10 to P20 (Supplementary Table 1). The disparity index and ratio were not affected in BDNF-PC-KO mice during postnatal development (Supplementary Table 2). These results indicate that BDNF derived from PCs is specifically involved in the late phase of CF elimination after P16.

### Altered PF and inhibitory inputs to PCs in BDNF-PC-KO mice

Analyses of several animal models have shown that normal synapse formation on PCs from PFs is prerequisite for the late phase of CF elimination^13^. We examined input–output relationships of PF-mediated EPSCs (PF-EPSCs) and found that PF-EPSC amplitudes at stimulus intensities over 5 μA were significantly larger in BDNF-PC-KO mice than those in control mice (Supplementary Fig. 5a, b). There was no significant difference in paired-pulse ratio of PF-EPSCs between the genotypes (Supplementary Fig. 5c, d). These results suggest that the lack of BDNF in postsynaptic PCs did not impair but rather enhanced PF-PC synaptic transmission. Thus, it is unlikely that the impaired CF synapse elimination in BDNF-PC-KO mice is caused secondarily by the impaired PF-PC synapse formation.

Our previous study demonstrates that GABAergic inhibition onto PCs regulates CF synapse elimination^39^. In mice with deletion of a single allele of the GABA synthesizing enzyme GAD67, CF synapse elimination is impaired after P10, which presumably results from reduced GABAergic inhibition from basket cells to PCs^39^. Because previous reports showed modulation of GABAergic inhibition by BDNF signaling^40, 41^, lack of BDNF may alter GABAergic inhibition onto PCs and may indirectly influence CF synapse elimination. To examine this possibility, we recorded miniature inhibitory postsynaptic currents (mIPSCs) from PCs at P15–P16^39^. The amplitude of mIPSCs in BDNF-PC-KO mice was similar to that in control mice (Supplementary Fig. 5e, f, *left*), but the frequency was markedly decreased in BDNF-PC-KO mice (Supplementary Fig. 5e, f, *right*). Furthermore, we found that the amplitude of IPSCs evoked (eIPSCs) by stimulating inhibitory inputs to PCs in the ML was significantly decreased (Supplementary Fig. 5l, m), whereas the paired-pulse ratio was unchanged, in BDNF-PC-KO mice (Supplementary Fig. 5l, m). These electrophysiological data indicate that the number of inhibitory synapses is significantly decreased in BDNF-PC-KO mice. This notion is consistent with our immunofluorescence data indicating that the density of parvalbumin (PA)-positive GABAergic interneurons in the ML (Supplementary Fig. 5g1, g2, i) and of vesicular inhibitory amino acid transporter-positive GABAergic terminals on the PC soma (Supplementary Fig. 5h, j) were significantly decreased, but the density of GABAergic terminals in the ML (Supplementary Fig. 5h, k) was unchanged, in BDNF-PC-KO mice. Thus, the possibility remains that the impaired CF synapse elimination in BDNF-PC-KO mice results from reduced GABAergic inhibition of PCs.

### BDNF derived from PCs is involved in CF synapse elimination

**Fig. 2:**
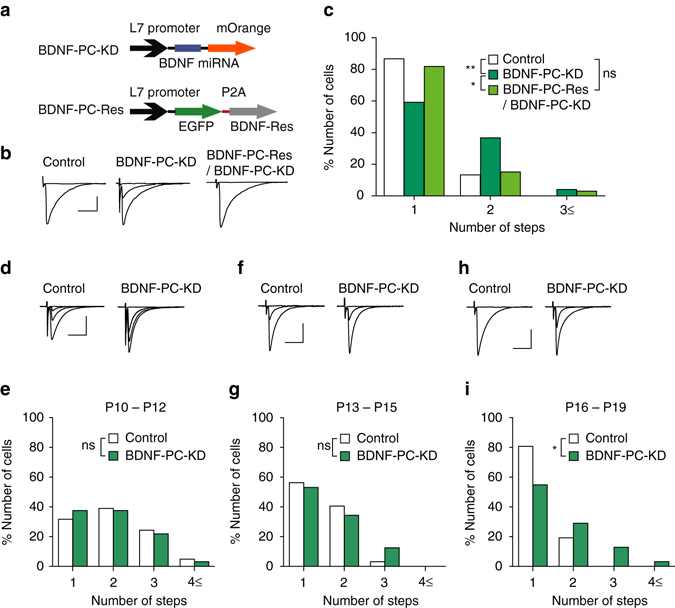
BDNF derived from postsynaptic PCs is involvement in CF synapse elimination. **a** Schema of vector constructs for BDNF-PC-KD and BDNF-PC-Res. **b** Representative traces of CF-EPSCs recorded in a PC from a control (*left*, P21), a BDNF-KD (*middle*, P21) and a BDNF-PC-Res/BDNF-PC-KD (*right*, P24) mouse. Holding potential was −10 mV. Single traces recorded at different stimulus intensities were superimposed. Scale bars, 10 ms and 1 nA. **c** Frequency distribution histogram for the number of CFs innervating each PC in control (*open columns*, *n* = 39 PCs from 11 mice), BDNF-KD (*green columns*, *n* = 49 PCs from 11 mice) and BDNF-Res (*light green columns*, *n* = 33 PCs from 3 mice) PCs during P21–P46. (*P* = 0.0036, control vs. BDNF-PC-KD; *P* = 0.038, BDNF-PC-KD vs. BDNF-PC-Res/BDNF-PC-KD; *P* = 0.509, control vs. BDNF-PC-Res/BDNF-PC-KD, Mann–Whitney *U*-test) *P < 0.05, **P < 0.01, ns indicates no significant difference. **d**–**i** Representative traces of CF-EPSCs (**d**, **f**, **h**) and frequency distribution histograms for the number of CFs innervating each PC (**e**, **g**, **i**) for control (*open columns*) and BDNF-KD (*green columns*) PCs during P10–P12 (**d**, **e**), P13–P15 (**f**, **g**), and P16–P19 (**h**, **i**). CF-EPSCs were recorded in cerebellar slices from a mouse at P11 (**d**), P13 (**f**), or P17 (**i**). Holding potential was −10 mV. Single traces recorded at different stimulus intensities were superimposed. Scale bars, 10 ms and 1 nA. Sample sizes for the frequency distribution histograms were 41 control and 32 BDNF-KD PCs from the same 5 mice for P10–P12 (*P* = 0.576, Mann–Whitney *U*-test) (**e**), 32 control and 32 BDNF-KD PCs from the same 4 mice for P13–P15 (*P* = 0.585, Mann–Whitney *U*-test) (**g**), and 26 control and 31 BDNF-KD PCs from the same 4 mice for P16–P19 (*P* = 0.025, Mann–Whitney *U*-test) (**i**). *P < 0.05; ns indicates no significant difference between the groups

It has been reported that the D2CreN line (GluD2^*+/cre*^) we used to generate BDNF-PC-KO mice expresses *cre* gene not only in PCs but also in ML interneurons^23, 42^. When mice with a floxed gene are crossed with the δ2CreN line, the targeted gene will be deleted in both PCs and molecular interneurons^23, 42, 43^. Therefore, BDNF in ML interneurons may also be deleted in BDNF-PC-KO mice. Hence, there remains a possibility that the phenotypes of BDNF-PC-KO mice might be partially attributed to the lack of BDNF from ML interneurons. We deleted BDNF specifically from PCs using lentiviral-mediated knockdown system^26, 27, 44^. We first confirmed the efficacy of BDNF knockdown by the microRNA (miRNA) vector using HEK 293T cells (Supplementary Fig. 6a, b). We injected lentivirus vectors expressing mOrange and miRNA for BDNF knockdown which were driven by the PC-specific L7 promoter into the mouse cerebellum at P1 (BDNF-PC-KD, Fig. 2a). We counted the number of CFs innervating mOrange-positive PCs with BDNF knockdown and that innervating mOrange-negative control PCs in the same slices (Fig. 2b, c). We found that the percentage of PCs innervated by two or more CFs were significantly higher in PCs with BDNF knockdown (BDNF-PC-KD) than in control PCs (Fig. 2c). We found no significant differences in the EPSC parameters for CF-mono, CF-multi-S and CF-multi-W (Supplementary Table 3), total CF-EPSC amplitude, disparity ratio or index (Supplementary Table 4) between control and BDNF-KD PCs, indicating normal strengthening of single “winner” CFs and their dendritic translocation.

To exclude possible off-target effect of the used miRNA, we constructed a miRNA-resistant form of BDNF tagged with EGFP driven by the L7 promoter (BDNF-PC-Res, Fig. 2a, c). The effect of BDNF-PC-KD was effectively rescued by coexpression of BDNF-PC-Res (Fig. 2c) to the same level as control PCs from BDNF-PC-KD slices (Fig. 2c). These results clarify that BDNF in postsynaptic PCs is involved in CF synapse elimination.

The effects of BDNF knockdown in PCs were not obvious at P10–P12 (Fig. 2d, e) and at P13–P15 (Fig. 2f, g), but BDNF-KD PCs were innervated by significantly higher number of CFs than control PCs at P16–P19 (Fig. 2h, i). Thus, we concluded that deletion of BDNF in PC is sufficient to cause the impairment of CF synapse elimination after P16.

**Fig. 3:**
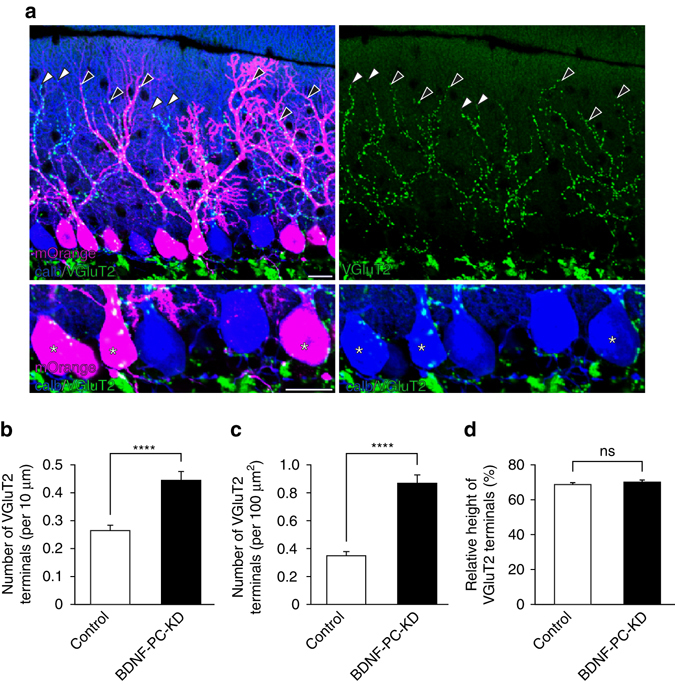
BDNF requirement for the elimination of CF synapses from the PC soma. **a** Triple fluorescent labeling for calbindin (*blue*), VGluT2 (*green*), and mOrange-positive BDNF-KD PCs (*magenta*) from a P21 mouse. Asterisks indicate the somata of mOrange-positive BDNF-KD PCs. *White* and *black arrowheads* indicate the most diatal tip of VGluT2-positive CF terminals along dendrites of control and BDNF-KD PCs, respectively. **b**, **c** Summary bar graphs showing the number of VGluT2-positive terminals per 10 μm membrane of the PC soma (**b**) and the number of VGluT2-positive terminals per 100 μm^2^ of the PC somatic area (**c**). Data were collected from mOrange-nagative control PCs (*n* = 283) and mOrange-positive BDNF-KD PCs (*n* = 241) from 3 mice (for **b**, control: 0.27 ± 0.02, BDNF-PC-KD: 0.44 ± 0.03, *P* < 0.0001; for **c**, control: 0.35 ± 0.03, BDNF-PC-KD: 0.87 ± 0.06, *P* < 0.0001, Mann–Whitney *U*-test). **d** Summary bar graph showing the relative height of CF terminals to the thickness of the ML for mOrange-negative control PCs (*n* = 108) and mOrange-positive BDNF-KD PCs (*n* = 108), from 3 mice (control: 68.9 ± 0.8, BDNF-PC-KD: 70.4 ± 0.9, *P* = 0.166, Mann–Whitney *U*-test). The distance between the most distal tip of VGluT2-posotive CF terminals along dendrites and the center of the soma was measured for each PC and the value was divided by the thickness of the ML. Values in **b**, **c** and **d** represent mean ± s.e.m. ns indicates no significant difference between the two groups. Scale bars, 20 μm

We next performed morphological examination of CF innervation of BDNF-KD PCs (Fig. 3a). We found the density of VGluT2-positive CF terminals on the PC soma was significantly higher in BDNF-KD PCs than in control PCs at P21 (Fig. 3b, c). In contrast, no significant difference was found in the relative height of VGluT2-positive CF terminals along PC dendrite between control and BDNF-KD PCs (Fig. 3d). We confirmed that expression of mOrange alone in PCs had no effect on CF innervation (Supplementary Fig. 7). These results indicate that BDNF KD in PCs specifically impairs elimination of somatic CF synapses without affecting dendritic translocation of “winner” CFs. To examine whether BDNF KD in PCs affects inputs from PFs and ML interneuron to PCs, we analyzed input–output relationships of PF-EPSCs, mIPSCs, and eIPSCs in BDNF KD PCs. We found no significant differences between BDNF-KD and control PCs for PF-EPSCs, mIPSCs, or eIPSCs (Supplementary Fig. 6c–h). These results strongly suggest that PC-derived BDNF contributes directly to CF synapse elimination, not indirectly through alteration of PF or inhibitory inputs to PCs.

### Neither TrkB nor p75^NTR^ in PCs mediates CF elimination

**Fig. 4:**
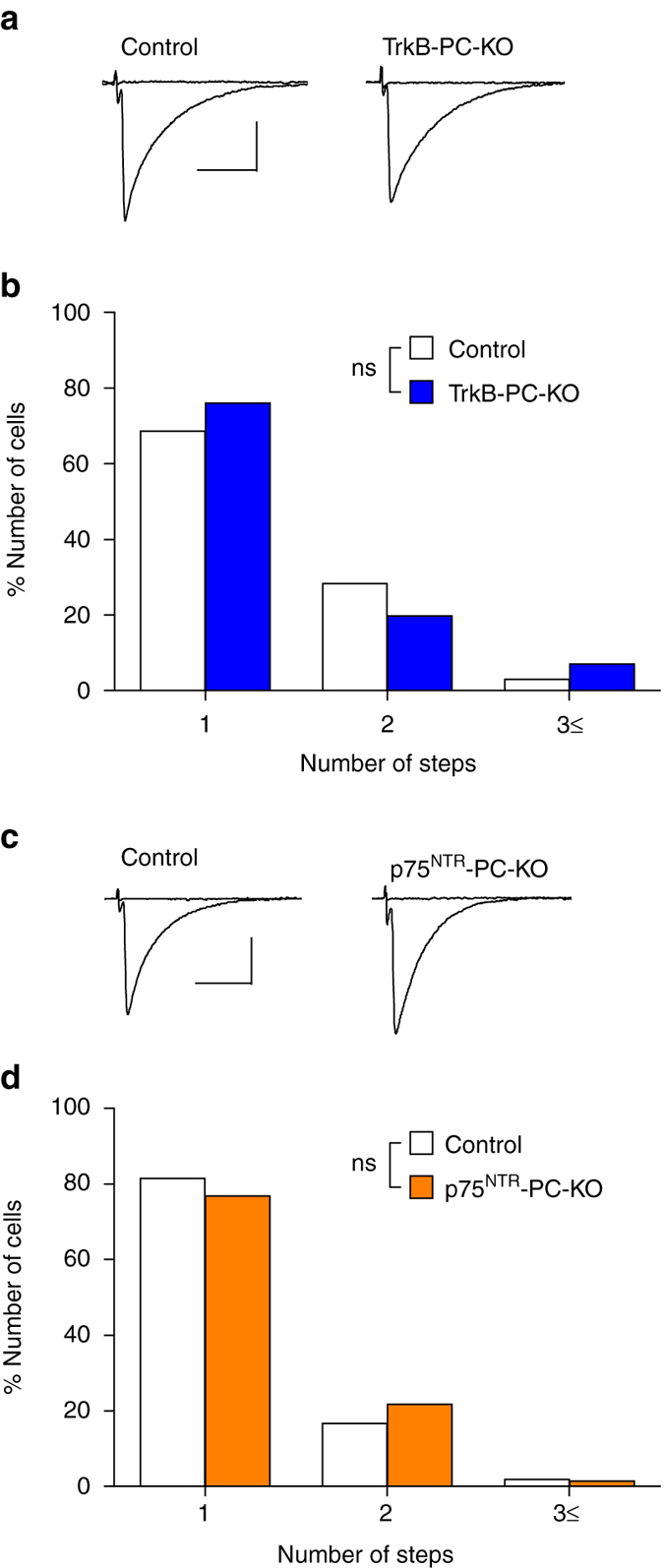
CF synapse elimination in TrkB-PC-KO and p75^NTR^-PC-KO mice. **a** Representative traces of CF-EPSCs in a PC from a control (*left*, P21) and a TrkB-PC-KO (*right*, P26) mouse. Holding potential was −10 mV. Single traces recorded at different stimulus intensities were superimposed. **b** Frequency distribution histogram for the number of CFs innervating each PC in control (*open columns*, *n* = 67 PCs from 7 mice) and TrkB-PC-KO (*filled columns*, *n* = 71 PCs from 7 mice) mice during P21–P31 (*P* = 0.612, Mann–Whitney *U*-test). ns indicates no significant difference between the two groups. **c**, **d** Similar to **a**, **b** but for results from p75^NTR^-PC-KO and control mice during P21–P41. CF-EPSCs in **c** were recorded in cerebellar slices from a control (*left*, P39) and a p75^NTR^-PC-KO (*right*, P38) mouse. Sample sizes for **d** were 54 PCs from 6 control mice and 69 PCs from 8 p75^NTR^-PC-KO mice (*P* = 0.548, Mann–Whitney *U*-test). ns indicates no significant difference between the two groups. Scale bars: **a** and **c**, 10 ms and 1 nA

To test whether BDNF derived from PCs acts in an autocrine manner, we generated conditional KO mice in which TrkB (*Ntrk2*) or p75^NTR^ (*Ngfr*) was deleted in PCs (Supplementary Fig. 8a–d). We confirmed the deletion of TrkB or p75^NTR^ only in genomic DNA prepared from the cerebellum of respective PC-specific KO mice (Supplementary Fig. 8e, f). We found no significant difference between control and TrkB-PC-KO mice in the number of CFs innervating each PC (Fig. 4a, b), basic EPSC parameters for CF-mono, CF-multi-S and CF-multi-W (Supplementary Table 5), total CF-EPSC amplitude, disparity ratio and index (Supplementary Table 6). Similarly, there was no significant difference between control and p75^NTR^-PC-KO mice in the number of CFs innervating each PC (Fig. 4c, d), basic parameters of CF-EPSCs (Supplementary Table 5), total CF-EPSC amplitude, disparity ratio and index (Supplementary Table 6). These results indicate that TrkB and p75^NTR^ in PCs are dispensable for BDNF signaling for CF synapse elimination.

### TrkB but not p75^NTR^ in CFs mediates CF elimination

**Fig. 5:**
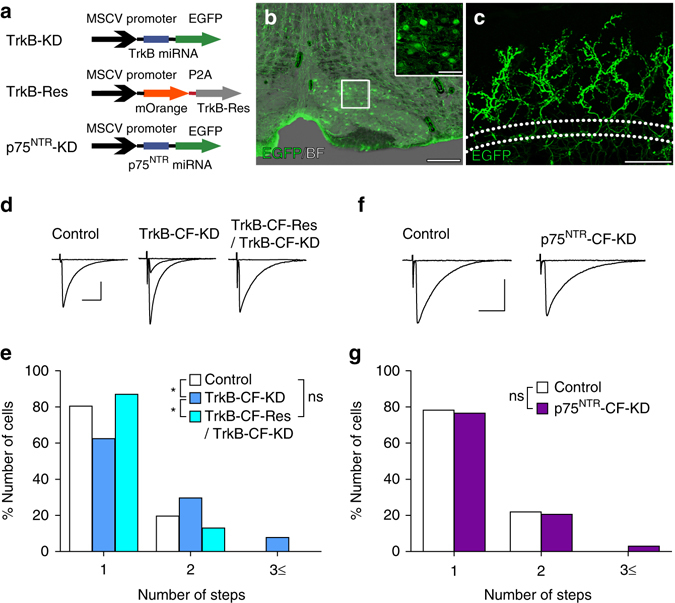
Effect of knockdown of TrkB or p75^NTR^ in inferior olivary neurons on CF synapse elimination. **a** Schema of vector constructs for TrkB-KD, TrkB-Res and p75^NTR^-KD. **b**, **c** Bright field (*BF*) and fluorescent images showing the expression of EGFP in inferior olivary neurons **b** and CFs in the cerebellum **c** from a P21 mouse. The *box* in **b** is enlarged in the inset. *Dotted lines* in **c** indicate the PC layer. Scale bars: **b**, 200 mm; **c**, 50 μm; inset in **b**, 20 μm. **d** Representative traces of CF-EPSCs recorded from a control PC (*left*, from a P21 mouse), a PC associated with TrkB-KD CFs (*middle*, from a P26 mouse) and a PC associated with CFs expressing both TrkB-KD and TrkB-Res (*right*, from a P21 mouse). Single traces recorded at different stimulus intensities were superimposed. Holding potential was −10 mV. Scale bars, 10 ms and 1 nA. **e** Frequency distribution histogram for the number of CFs innervating each PC for control PCs (*open columns*, *n* = 46 PCs from 11 mice), PCs associated with TrkB-KD CFs (*blue columns*, *n* = 64 PCs from 11 mice) and PCs associated with CFs expressing both TrkB-KD and TrkB-Res (*light blue columns*, *n* = 23 PCs from 3 mice) during P21–P59. (*P* = 0.0303, control vs. TrkB-CF-KD; *P* = 0.026, TrkB-CF-KD vs. TrkB-CF-Res/TrkB-CF-KD; *P* = 0.509, control vs. TrkB-CF-Res/TrkB-CF-KD, Mann–Whitney *U*-test). Control PCs and PCs associated with TrkB-KD CFs were recorded in the same mice. *P < 0.05. **f**, **g** Similar to **d**, **e** but for results from PCs associated with p75^NTR^-KD CFs and control PCs during P22–P25. CF-EPSCs in **f** were recorded in cerebellar slices from a control PC (left, from a P21 mouse) and a PC associated with p75^NTR^-KD CFs (*right*, from a P21 mouse). Scale bars, 10 ms and 1 nA. Sample sizes for **g** were 32 control PCs and 34 PCs associated with p75^NTR^-KD CFs in the same 5 mice (*P* = 0.832, Mann–Whitney *U*-test). ns indicates no significant difference between the groups

We next examined the possibility that BDNF acts retrogradely from PCs to CFs by knocking down TrkB or p75^NTR^ in CFs. We generated lentiviral vectors carrying miRNA against TrkB or p75^NTR^ tagged with EGFP driven by the murine stem cell virus (MSCV) (TrkB-KD, p75^NTR^-KD, Fig. 5a, Supplementary Fig. 9a–d). We injected the lentiviral KD vectors into the inferior olive of mice at P1^27^, which resulted in successful transfection of the vectors in a certain proportion of inferior olivary neurons (Fig. 5b). We prepared cerebellar slices from the mice during P21–P59 and recorded CF-EPSCs from PCs in cerebellar regions where most PC somata were surrounded by EGFP-positive CFs (TrkB or p75^NTR^-KD CFs, Fig. 5c). As control, we also recorded CF-EPSCs from PCs in the same cerebellar slices where EGFP-positive CFs were absent. In PCs associated with TrkB-KD CFs, 37.50% were innervated by two or more CFs, whereas 19.57% did so in control PCs (Fig. 5d, e). The effect of TrkB knockdown in CFs was rescued by co-expression of the TrkB miRNA-resistant form of TrkB tagged with mOrange driven by the MSCV (TrkB-Res) (Fig. 5d, e). In marked contrast, there was no difference in the number of CFs between PCs surrounded by p75^NTR^-KD CFs and control PCs (Fig. 5f, g). We found that neither TrkB-KD nor p75^NTR^-KD in CFs affected basic EPSC parameters for CF-mono, CF-multi-S and CF-multi-W (Supplementary Table 7), total CF-EPSC amplitude, disparity ratio and index (Supplementary Table 8) in PCs surrounded by CFs with TrkB or p75^NTR^–KD. Moreover, TrkB KD in CFs did not affect input–output relationship of PF-EPSCs or the amplitude and frequency of mIPSCs (Supplementary Fig. 10a–d). Together, these results strongly suggest that TrkB but not p75^NTR^ in CFs mediates the action of BDNF derived from PCs for CF synapse elimination.

### TrkB in CFs mediates the effect of BDNF derived from PCs

**Fig. 6:**
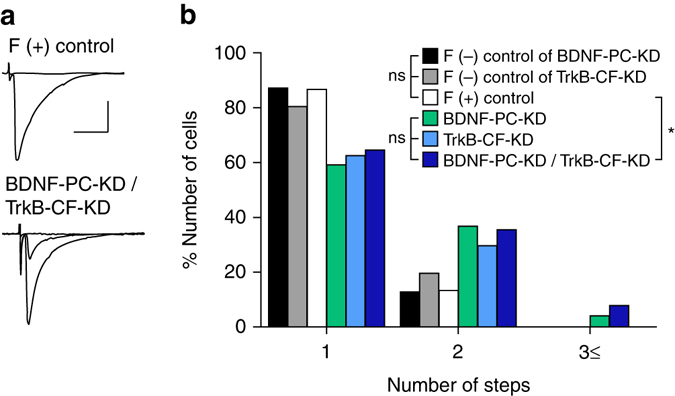
BDNF from PCs acts on TrkB on CFs during CF synapse elimination. **a** Representative traces of CF-EPSCs recorded in a mOrange-positive control PC associated with EGFP-positive control CFs (*top*, from a P21 mouse) and a mOrange-positive BDNF-KD PC associated with EGFP–positive TrkB-KD CFs (*bottom*, from a P34 mouse). Scale bars, 10 ms and 1 nA. **b** Frequency distribution histograms for the number of CFs innervating each PC for the following six groups of PCs recorded in mice during P21–P46. (1) F(−) control of BDNF-PC-KD: fluorescence-negative control PCs sampled in the same slices for sampling BDNF-KD PCs (*closed columns*, *n* = 39 PCs from 11 mice); (2) F(−) control of TrkB-CF-KD: fluorescence-negative control PCs that were not associated with TrkB-KD CFs (*gray columns*, *n* = 46 PCs from 11 mice); (3) F(+) control: mOrange-positive control PCs associated with EGFP–positive control CFs (*open columns*, *n* = 30 PCs from 4 mice); (4) BDNF-PC-KD: PCs with BDNF-KD sampled in the same slices for sampling F(−) control PCs (*green columns*, *n* = 49 PCs from 11 mice), (5) TrkB-CF-KD: PCs associated with TrkB-KD CFs (*blue columns*, *n* = 64 PCs from 11 mice); (6) BDNF-PC-KD/TrkB-CF-KD: BDNF-KD PCs associated with TrkB-KD CFs (*deep blue columns*, *n* = 31 PCs from 5 mice). *P* = 0.643 among the three control groups (Kruskal–Wallis test with Dunn’s post-hoc test). *P* = 0.838 among the three KD groups (Kruskal–Wallis test with Dunn’s post-hoc test). *P* = 0.047 between F(+) control and BDNF-PC-KD/TrkB-CF-KD (Mann–Whitney *U*-test). F(+), fluorescent protein-positive; F(−), fluorescent protein-negative *P < 0.05, ns indicates no significant difference among the three groups

To obtain further evidence that BDNF from PCs exerts its effect through TrkB in CFs, we conducted simultaneous KD of BDNF in PCs and TrkB in CFs and compared the effect of the double KD and that of single KD of either molecule. We first confirmed that expression of mOrange only and EGFP only into PCs and CFs, respectively (F (+) controls in Fig. 6a, b), had no effect on CF innervation when compared with fluorescent protein-negative control PCs (*black*, *gray*, and *open columns* in Fig. 6b). We found that significantly higher percentage of PCs were innervated by multiple CFs in BDNF-PC-KD/TrkB-CF-KD than in fluorescent protein-positive (F (+)) control (*open* vs. *deep blue columns* in Fig. 6b). However, there was no difference in the percentage of multiply innervated PCs among single BDNF-PC-KD, single TrkB-CF-KD, and BDNF-PC-KD/TrkB-CF-KD (*green*, *blue* and *deep blue columns* in Fig. 6b). These data demonstrate that the effect of TrkB-KD in CFs on CF synapse elimination was occluded by BDNF-KD in PCs, and therefore indicate that TrkB in CFs mediates the effect of BDNF derived from PCs on CF synapse elimination. Taken all together, our present results indicate that BDNF to TrKB signaling does not contribute to functional differentiation, CF translocation, or early phase of CF elimination, but is specifically involved in the final stage of the late phase of CF elimination after P16 (Supplementary Fig. 11).

As shown in Fig. 3, deletion of BDNF in PCs impairs elimination of CF synapses from the PC soma without affecting CF translocation to PC dendrites. This data suggest the possibility that TrkB is more strongly expressed in weaker CFs innervating PC somata than single winner CFs extending their innervation to PC dendrites. To test this possibility, we performed immunohistochemical analyses of TrkB expression in CFs on PC somata and those on PC dendrites in the cerebellum at P12 when the strongest CFs are translocating to PC dendrites and synaptic terminals of weaker CFs are present on the PC soma. We first confirmed the specificity of the TrkB antibody by comparing the intensities of immunostaining signals from PC somata between wild-type and TrkB-PC-KO mice (Supplementary Fig. 12). We found that TrkB immunofluorescence signals were present diffusely in the ML and GL (Supplementary Fig. 13a, c). In addition, we detected clear TrkB expressions in CF terminals on both somata (Supplementary Fig. 13c–e) and dendrites (Supplementary Fig. 13a, b) of PCs. However it was impossible to detect a clear difference in the intensity of TrkB signals between somatic and dendritic CF terminals. Therefore, it remains unclear whether the differential effect of BDNF deletion from PCs on somatic and dendritic CFs results from the differential levels of TrkB expression in CFs.

### BDNF, Sema7A, and mGlu1 share a common signaling pathway

**Fig. 7:**
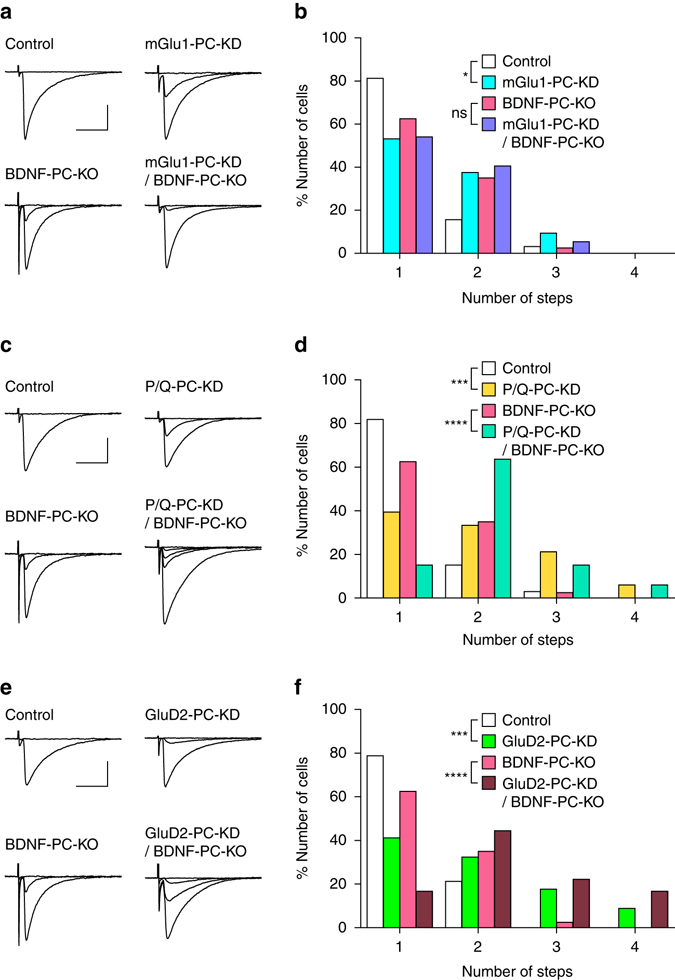
BDNF and mGlu1 share a common signaling pathway for CF synapse elimination. **a** Representative CF-EPSC traces for control and mGlu1-PC-KD (from a P24 wild-type mouse) and for BDNF-PC-KO and mGlu1-PC-KD/BDNF-PC-KO (from a P24 BDNF-PC-KO mouse). Holding potential was −10 mV. Single traces recorded at different stimulus intensities were superimposed. **b** Frequency distribution histogram for the number of CFs innervating each PC showing the effects of mGlu1-knockdown in PCs of wild-type mice (Control, *n* = 32; mGlu1-PC-KD, *n* = 32, from 4 wild-type mice during P21–P35, *P* = 0.018, Mann–Whitney *U*-test) and in PCs of BDNF-PC-KO mice (BDNF-PC-KO, *n* = 40; mGlu1-PC-KD/BDNF-PC-KO, *n* = 37, from 4 BDNF-PC-KO mice during P21–P35, *P* = 0.419, Mann–Whitney *U*-test). **c**, **d** Similar to **a**, **b** but for the effects of P/Q-VDCC knockdown in PCs of wild-type mice (Control, *n* = 32 PCs; P/Q-PC-KD, *n* = 33 PCs, from 5 wild-type mice during P21–P35, *P* = 3.0 × 10^−4^, Mann–Whitney *U*-test) and in PCs of BDNF-PC-KO mice (BDNF-PC-KO, *n* = 40 PCs; P/Q-PC-KD/BDNF-PC-KO, *n* = 33 PCs, from 4 BDNF-PC-KO mice during P21–P35, *P* < 0.0001, Mann–Whitney *U*-test). CF-EPSCs in **c** were from a P35 wild-type mouse (Control and P/Q-PC-KD) and from a P36 BDNF-PC-KO mouse (BDNF-PC-KO and P/Q-PC-KD/BDNF-PC-KO). **e**, **f** Similar to **a**, **b** and **c**, **d** but for the effects of GluD2 knockdown in PCs of wild-type mice (Control, *n* = 32 PCs; GluD2-PC-KD, *n* = 34 PCs, from 4 wild-type mice during P21–P35, *P* = 5.0 × 10^−4^, Mann–Whitney U-test) and in PCs of BDNF-PC-KO mice (BDNF-PC-KO, *n* = 40 PCs; GluD2-PC-KD/BDNF-PC-KO, *n* = 36 PCs, from 4 BDNF-PC-KO mice during P21–P35, *P* < 0.0001, Mann–Whitney *U*-test). CF-EPSCs in **e** were from a P23 wild-type mouse (Control and GluD2-PC-KD) and from a P36 BDNF-PC-KO mouse (BDNF-PC-KO and P/Q-PC-KD/BDNF-PC-KO). Scale bars, **a**, **c**, and **e**, 10 ms and 1 nA. *P < 0.05, ***P < 0.001, ****P < 0.0001. ns indicates no significant difference between the groups

Previous studies have shown that mGlu1, P/Q-VDCC, and GluD2 in PCs are crucial for different aspects of CF synapse elimination^15, 20, 22, 23, 45^. We thus examined whether the BDNF signaling for CF synapse elimination is along the same signaling cascade of mGlu1, P/Q-VDCC, or GluD2. First, we confirmed that knockdown of mGlu1, P/Q-VDCC, or GluD2 in wild-type PCs impaired CF synapse elimination (*open* vs. *blue columns* in Fig. 7a, b; *open* vs. *yellow columns* in Fig. 7c, d; *open* vs. *green columns* in Fig. 7e, f)^15, 23, 27^. We then examined the effect of knockdown of mGlu1, P/Q-VDCC, or GluD2 in PCs of BDNF-PC-KO mice. We found no significant difference in the number of CFs innervating each PC between control PCs and mGlu1-knockdown PCs in BDNF-PC-KO mice (*pink* vs. *violet columns* in Fig. 7b). In contrast, knockdown of either P/Q-VDCC or GluD2 in PCs of BDNF-PC-KO mice caused more severe impairment of CF synapse elimination than respective control PCs in BDNF-PC-KO mice (P/Q-VDCC, *pink* vs. *green columns* in Fig. 7d; GluD2, *pink* vs. *brown columns* in Fig. 7f). These results suggest that BDNF shares a signaling pathway for CF synapse elimination with mGlu1, but not with P/Q-VDCC or GluD2.

**Fig. 8:**
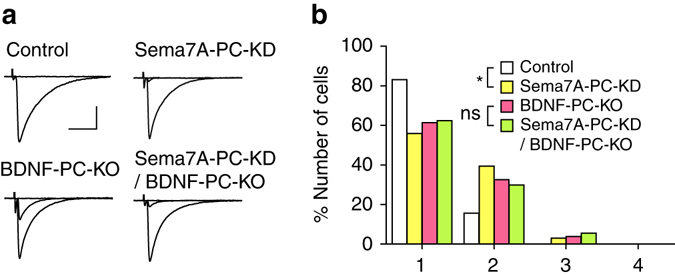
BDNF and Sema7A share a common signaling pathway for CF synapse elimination. **a** Representative traces of CF-EPSCs for control and Sema7A-PC-KD were recorded from a P21 control mouse, and those for BDNF-PC-KO and Sema7A-PC-KD/BDNF-PC-KO were from a P22 BDNF-PC-KO mouse. Holding potential was −10 mV. Single traces recorded at different stimulus intensities were superimposed. Scale bars, 10 ms and 1 nA. **b** Frequency distribution histogram for the number of CFs innervating each PC showing the effects of Sema7A-knockdown in PCs of control mice (Control, *n* = 31; Sema7A-PC-KD, *n* = 30, from 5 control mice during P21–P25, *P* = 0.022, Mann–Whitney *U*-test) and in PCs of BDNF-PC-KO mice (BDNF-PC-KO, *n* = 45; Sema7A-PC-KD/BDNF-PC-KO, *n* = 49, from 6 BDNF-PC-KO mice during P21–P26, *P* = 0.975, Mann–Whitney *U*-test). *P < 0.05, ns indicates no significant difference between the groups

We have reported previously that a membrane-anchored semaphorin, Sema7A, mediates retrograde signaling for CF synapse elimination downstream of mGlu1^27^. We thus examined whether BDNF shares the same signaling with Sema7A for CF synapse elimination^27^. First, we confirmed that Sema7A knockdown in PCs of control mice impaired CF synapse elimination (*open* vs. *yellow columns* in Fig. 8a, b)^27^. In contrast, we found no significant difference in the number of CFs innervating each PC between EGFP-positive Sema7A knockdown PCs and EGFP-negative control PCs in BDNF-PC-KO mice (*pink* vs. *green columns* in Fig. 8a, b), indicating that the effect of Sema7A knockdown was occluded in PCs with BDNF deletion. We therefore conclude that BDNF and Sema7A share the same signaling for CF synapse elimination.

## Discussion

We have shown that PC-derived BDNF retrogradely acts on TrkB in CFs and promotes their elimination in the developing cerebellum. Since BDNF to TrkB signaling generally support neuronal survival, development, maintenance of innervating neurons, synapse formation, and synaptic potentiation^28^, our present findings are apparently contrary to the widely accepted actions of BDNF to TrkB signaling. Previous studies showed that developmental CF synapse elimination is impaired in global TrkB knockout mice^32^ and in mice with conditional deletion of TrkB from the cerebellum^33^. Our present results are consistent with these studies and clarified the cellular localization of TrkB and the identity and the origin of its agonist BDNF. We have also found that CF synapse elimination does not require p75^NTR^ that is known to mediate multiple cellular responses including neurite growth, cell death and survival/growth differentiation depending on the receptors and intracellular signaling protein with which it interacts^46^. Notably, p75^NTR^ have been reported to mediate developmental axon pruning in sympathetic neurons projecting eyes^47^ and retraction of less active motor neuron axons in the developing neuromuscular junction^48, 49^. These results indicate that CF synapse elimination in the developing cerebellum is a unique cellular process in which BDNF mediate punishment rather than reward signals through TrkB.

In the neuromuscular junction, BDNF has been reported to be involved in developmental axon pruning. Je et al. has reported that postsynaptic muscle cells releases the precursor of BDNF (pro BDNF) in an activity-dependent manner, and that proteolytic conversion of proBDNF to mature BDNF occurs around active presynaptic terminals of motoneurons, presumably because of activity-dependent release of protease from presynaptic terminals. Then, mature BDNF selectively stabilizes active nerve terminals through TrkB, whereas proBDNF causes retraction of less active nerve terminals through p75^NTR^ ^48, 49^. The authors conclude that mature BDNF and proBDNF function as reward and punishment signals for synapse elimination at developing neuromuscular junction through TrkB and p75^NTR^, respectively^48, 49^. By analogy to the action of BDNF in the developing neuromuscular junction, it is logically possible that BDNF is involved in cerebellar CF synapse elimination as a supporting signal. One possibility would be that BDNF strengthens CF synaptic inputs and enhances the functional differentiation into a single winner CF and several loser CFs in each PC. Then, the strongest winner CF synaptic inputs would generate punishment signals that overcome the supporting effects of BDNF on weaker CF synapses and eventually eliminate them from the PC soma. However, this possibility is unlikely, since the amplitude and kinetics of EPSCs elicited by the strongest CFs were not altered by deletion of BDNF from PCs. Our present results suggest that BDNF and TrkB are used differently for developmental synapse elimination between the neuromuscular junction and the cerebellar CF to PC synapse, although the two systems are thought to share the basic mechanisms including activity dependency, homosynaptic competition and the ‘winner-takes-all’ rule. In the developing cerebellum, CF to PC synapse elimination involves not only homosynaptic competition among multiple CFs innervating the same PC but also heterosynaptic competition between PF inputs and CF synapses^7, 13, 20, 21^. In addition, GABAergic inhibition plays an important role for the control of CF synapse elimination^39^. It should also be noted that four distinct phases are present in CF synapse elimination^7, 8^. Presumably because of such complexity, CF to PC synapse elimination would require multiple signaling cascades that are not involved in axon pruning at the neuromuscular junction. Hence, the role played by BDNF to TrkB signaling would be more specialized and limited in CF synapse elimination than in axon pruning at the neuromuscular junction.

We found that BDNF to TrKB signaling did not influence the selection of a single”winner” CF and its dendritic translocation. Furthermore, deletion of BDNF to TrkB signaling did not affect the amplitudes of EPSCs for CF-mono and CF-multi-S. These results are consistent with the previous reports of TrkB-KO mice^32, 33^, and indicate that BDNF does not act as a trophic factor for CF to PC synapses during normal development of the cerebellum. However, after surgical transection of olivo-cerebellar axons at P3, regrowth of olivary axons and synaptogenesis from CFs to PCs has been reported to involve BDNF to TrkB signaling^50^. This result suggests that BDNF has potential to promote neurite outgrowth and synaptogenesis through TrkB under extreme conditions such as total transaction of unilateral olivo-cerebellar axons.

In contrast to the lack of BDNF effect during the first two postnatal weeks, we demonstrated that BDNF to TrKB signaling was required for the elimination of CF synapses from the PC soma after P16, indicating that PC-derived BDNF mediates punishment signal through TrKB on CFs specifically after P16. We found that the effect of mGlu1 knockdown in PCs, but not P/Q-VDCC or GluD2 knockdown in PCs, was occluded in BDNF-KO PCs, suggesting that BDNF to TrkB signaling is along the same pathway of mGlu1 cascade in PCs. How BDNF is released following mGlu1 activation remains to be elucidated. Previous studies have shown that secretion of BDNF from neurons is most pronounced under high-frequency stimulation or theta-burst stimulation^51, 52^. Since high-frequency burst of PF inputs effectively activates mGlu1 in PCs^53–55^, BDNF can be secreted from PCs following mGlu1 activation by PF synaptic inputs. Indeed, Furutani et al., have shown evidence that BDNF is secreted from PCs of mice at P21–P30 following mGlu1 activation and subsequent Ca^2+^ mobilization from internal stores through inositol 1,4,5-trisphosphate (IP_3_) receptors^56^.

Earlier studies on BDNF or TrkB KO mice have shown that axonal and dendritic arbolization of PCs and granule cells are moderately reduced in the absence of TrkB^57, 58^ or BDNF^59, 60^, whereas gross morphology of the cerebellum and the total number of PCs and granule cells are not altered. In addition, the density of GABAergic synapses is markedly reduced, whereas the morphology of glutamatergic synapses appears normal, in the cerebellum-specific TrkB KO mice^57^. In the present study, we found that PF to PC synaptic transmission was functionally strengthened, whereas the frequency of mIPSCs, the amplitude of eIPSCs and the density of GABAergic synapses were reduced, in PCs of BDNF-PC-KO mice. Gross anatomy of the cerebellum, morphology of PCs and Bergmann glia, and distribution of CF and PF synaptic terminals were normal in BDNF-PC-KO mice, which is consistent with the results of TrkB KO mice^32^. In contrast, we found that functional PF to PC synaptic strength, the frequency and amplitude of mIPSCs, the amplitude and paired-pulse ratio of eIPSCs, and the density of GABAergic synapses were normal in PCs with BDNF knockdown and in PCs associated with CFs with TrkB knockdown. These results indicate that the enhanced PF to PC synaptic strength and the reduced GABAergic inhibition in PCs of BDNF-PC-KO mice are attributable to the lack of BDNF in neurons other than PCs, presumably GABAergic interneurons in the ML. This is because the D2CreN line causes deletion of the targeted gene in both PCs and ML interneurons^42^. We therefore conclude that the impaired CF synapse elimination described in the present study was caused by the deletion of PC-derived BDNF, not secondarily by altered PF to PC excitatory inputs or reduced inhibition onto PCs. Thus, our present results present a unique action of BDNF as a punishment signal for developmental synapse elimination through TrkB from P16 to P20. A previous study suggests that BDNF derived from PCs is required for the maintenance of presynaptic function of PF-PC synapses but has no action on CF-PC synapses in mice from P23 to P29^56^. It remains to be investigated how the action and target PC-derived BDNF switches from punishment of somatic CF synapses to maintenance of PF inputs after the completion of CF synapse elimination. It also remains to be investigated whether BDNF exerts a similar action of punishment for developmental synapse elimination in other brain regions such as the retino-geniculate synapse that uses mGlu1 for the maintenance of its connectivity after synapse elimination^61^.

## Methods

### Animals

Experiments were approved by Experimental Animal Ethics Committees of The University of Tokyo, Hokkaido University, and Niigata University and were conducted according to the guidelines of the Japan Neuroscience Society. We generated the floxed mice carrying two loxP sequences in each genomic region of BDNF (*Bdnf*), TrkB (*Ntrk2*), and p75^NTR^ (*Ngfr*) (Supplementary Figs. 2 and 8). We generated each of conditional BDNF, TrkB and p75^NTR^ KO mice (BDNF-PC-KO, TrkB-PC-KO, and p75^NTR^-PC-KO) by intercrossing these floxed mice with a D2CreN line (GluD2^*+/Cre*^) whose *cre* gene was driven under the control of the GluD2 promotor^42^. All the KO mice utilized have C57BL/6 genetic background. The KO mice (both sexes, P0–P50 of age) and wild-type C57BL/6N mice (both sexes, P0–P59 of age, SLC Japan) were used for viral injections and the following electrophysiological experiments. All the experiments using KO mice and their littermates were performed in blind to mouse genotypes that were later identified by PCR of tail or finger samples using the following primers:

BD-KOF5′-AGGGAAGCCACAGTGTTC-3′

BD-lox35′-CTATCTTCCCCTTTTAATGG-3′

BD-lox25′-GAACGAAGATACTTAGTGTC-3′

TrkB-KOF5′-CTCTTAAGTCTCTAGCCTGG-3′

TrkB-loxR5′-TTTACCTCAGCTGCTAGGGC-3′

P75-KOF5′-CCAGCCATTCTGGATATCGG-3′

P75-loxR5′-CTCCACTCTGCAGTTTCCAC-3′

### Viral vector constructs

As described previously^26, 27, 44^, we designed VSV-G pseudotyped lentiviral vectors (pCL20c)^62^ for PC-specific expression under the control of a truncated L7 promoter (pCL20c-L7)^63^. For expression into inferior olivary neurons, we used the MSCV (pCL20c-MSCV) promoter. For vector-based RNA interference (RNAi) analysis, we used a BLOCK-iT Pol II miR RNAi expression vector kit (K4935-00, Invitrogen, CA, USA). The following engineered miRNAs were designed according to the BLOCK-iT Pol II miR RNAi Expression Vector kit guidelines (Invitrogen):

5′-TGCTGAAGTGTACAAGTCCGCGTCCTGTTTTGGCCACTGACTGACAGGACGCGCTTGTACACTT-3′ and

5′-CCTGAAGTGTACAAGCGCGTCCTGTCAGTCAGTGGCCAAAACAGGACGCGGACTTGTACACTTC-3′ for BDNF miRNA;

5′-TGCTGTTAGGTTCCAACCTCGGGAATGTTTTGGCCACTGACTGACATTCCCGATTGGAACCTAA-3′ and

5′-CCTGTTAGGTTCCAATCGGGAATGTCAGTCAGTGGCCAAAACATTCCCGAGGTTGGAACCTAAC-3′ for TrkB miRNA;

5′-TGCTGTAGACCTTGTGATCCATCGGCGTTTTGGCCACTGACTGACGCCGATGGCACAAGGTCTA-3′ and

5′-CCTGTAGACCTTGTGCCATCGGCGTCAGTCAGTGGCCAAAACGCCGATGGATCACAAGGTCTAC-3′ for p75^NTR^ miRNA;

5′-TGCTGAAATCAGGGAGTCTCTGATGAGTTTTGGCCACTGACTGACTCATCAGACTCCCTGATTT-3′ and

5′-CCTGAAATCAGGGAGTCTGATGAGTCAGTCAGTGGCCAAAACTCATCAGAGACTCCCTGATTTC-3′ for mGlu1 miRNA;

5′-TGCTGTTCCAATGAAGTATGGTTCCGGTTTTGGCCACTGACTGACCGGAACC ACTTCATTGGAA-3′ and

5′-CCTGTT CCAATGAAGTGGTTCCGGTCAGTCAGTGGCCAAAACCGGAACCATACTTCATTGGAAC-3′ for P/Q-VDCC miRNA;

5′-TGCTGTATGCATGGCATCTGCCAAAGGTTTTGGCCACTGACTGACCTTTGGC ATGCCATGCATA-3′ and

5′-CCTGTATGCATGGCATGCCAAAGGTCAGTCAGTGGCCAAAACCTTTGGCAG ATGCCATGCATAC-3′ for GluD2 miRNA. These oligonucleotides were subcloned into a pCL20c-trL7 vector or into a pCL20c-MSCV vector at 5′- or 3′-side of a fluorescent protein.

The cDNAs for BDNF, TrkB, and p75^NTR^ were obtained by RT-PCR of a cDNA library from P21 mouse cerebellum or medulla. We used the following PCR primers. BDNF cDNA, 5′-TGAGTCTCCAGGACAGCAAA-3′ and 5′-CTGTTTCCTTTCAGGTC

ATGG-3′; TrkB cDNA, 5′-ACTCCGACTGACTGGCACTG-3′ and 5′-TCCTGGTCCTG

TCAACACTG-3′; and p75^NTR^ cDNA, 5′-GCGGACTGAGCTAGAAGCTG-3′ and 5′-GAGG

CCCTACACAGAGATGC-3′. The Quik Change Lightning site-directed mutagenesis kit (Agilent Technologies, CA, USA) was used to generate RNAi-resistant forms of BDNF and TrkB (BDNF-Res, TrkB-Res), which harbor sense mutations (no alteration of amino acid codons). The following sequences were used to generate BDNF-res and TrkB-res:

5′-CCAGAAGGTTCGGCCCAACGAAGAAAACCATAAAGATGCTGATTTATATACATCCCGGGTGATGCTCAG-3′ and

5′-CTGAGCATCACCCGGGATGTATATAAATCAGCATCTTTATGGTTTTCTTCGTTGGGCCGAACCTTCTGG-3′ for BDNF-Res;

5′-CCTTCTCCAGGCATCGTGGCTTTGCCAAGATTAGAGCCAAACAGCGTTGACCCGGAGAA-3′ and

5′-TTCTCCGGGTCAACGCTGTTTGGCTCTAATCTTGGCAAAGCCACGATGCCTGGAGAAGG-3′ for TrkB-Res.

BDNF-Res and TrkB-Res were linked in-frame to mOrange2 and EGFP, respectively, which was interposed by a picornavirus “self-cleaving” P2A peptide sequence to enable efficient bicistronic expression. Each cDNA was subcloned into pCL20c-trL7 for BDNF-Res or pCL20c-MSCV for TrkB-Res. All constructs were verified by DNA sequencing.

### Lentiviral injection in vivo

For in vivo virus infection into the cerebellum or the inferior olive, a 33-Gauge Hamilton syringe filled with viral solution was attached to a micropump (UltramicroPump II, World Precision Instruments (WPI)). Under anesthesia by inhalation of isoflurane (1–2%), 4–5 μl (4–5 × 10^5^ TU) of viral solution was injected at a rate of 200 nl/min using a microprocessor-based controller (Micro4, WPI) into the cerebellar vermis of C57BL/6 mice at P0–1. For the inferior olive, 2–3 µl (1–2 × 10^5^ TU) of viral solution was injected at a rate of 100 nl/min into the ventral medial portion of the medulla of C57BL/6 mice at P1 under isoflurane anesthesia. The syringe was left for an additional 2 min before it was withdrawn. The scalp was then sutured and the mouse was returned to its home cage.

### Evaluation of knockdown efficacy

Human embryonic kidney (HEK) 293T cells in a 24-well dish were transfected with an RNAi knockdown vector (BDNF-KD, TrkB-KD or p75^NTR^-KD) and cDNA (BDNF, TrkB, p75^NTR^) using X-treme GENE 9 reagents (#06365787001, Roche) with 50 ng of vectors as indicated in Supplementary Figs. 6 and 9. One day later, the cells were fixed. After permeabilization, blocking, and application of the following primary and secondary antibodies, fluorescence signals were examined under a confocal laser scanning microscope (FV1200, Olympus). The primary antibodies used for the assays were the guinea pig anti-BDNF (raised against 131–249 amino acid residues (AY057908.1) by M. Watanabe, 1:400) and rabbit anti-DsRed (#632496, 1:1000, Clontech) antibodies for Supplementary Fig. 6a, the rabbit anti-TrkB (SAB4502034, 1:1000, Sigma) and rat anti-GFP (#06083-05, 1:1000, Nacalai) antibodies for Supplementary Fig. 9a, and the rabbit anti-p75^NTR^ ((N3908, 1:1000, Sigma) and rat anti-GFP (#06083-05, 1:1000, Nacalai) antibodies for Supplementary Fig. 9c. The secondary antibodies used for the assays were the donkey anti-guinea pig Alexa Flour 647 antibody (1:300, Jackson), the donkey anti-rabbit Cy3 antibody (1:300, Jackson), and the donkey anti-rat Alexa Flour 488 antibody (1:300, Jackson).

### Electrophysiology

Mice aged 10–52 days postnatally were decapitated following CO_2_ anesthesia, and brains were rapidly removed and placed into chilled external solution (0–4 °C) containing 125 mM NaCl, 2.5 mM KCl, 2 mM CaCl_2_, 1 mM MgSO_4_, 1.25 mM NaH_2_PO_4_, 26 mM NHCO_3_, and 20 mM glucose bubbled with 95% O_2_ and 5% CO_2_ (pH 7.4). Parasagittal cerebellar slices (250 μm thick) were prepared by using a vibratome slicer (VT1200s, Leica). The slices were recovered for 1 h at room temperature by incubating in a reservoir chamber bathed in the external solution. One slice was transferred to a recording chamber located on the stage of an upright microscope (BX51WI, Olympus). The recording chamber was continuously perfused with the oxygenated external solution supplemented with picrotoxin (0.1 mM, TOCRIS) to block inhibitory synaptic transmission. Whole-cell recordings were made from visually identified or fluorescent protein-positive PCs using an upright and fluorescent microscopes at 32 °C. The resistance of the patch pipettes was 1.5–2.5 MΩ when filled with the intracellular solution composed of 60 mM CsCl, 10 mM Cs d-Gluconate, 20 mM TEA-Cl, 20 mM BAPTA, 4 mM MgCl_2_, 4 mM Na_2_-ATP, and 0.4 mM Na_2_-GTP (pH 7.3, adjusted with CsOH). The pipette access resistance was compensated by 70%. For recording mIPSCs, 10 μM NBQX, 5 μM R-CPP, and 1 μM TTX (tetrodotoxin) were added. Ionic currents were recorded with an EPC9 patch clamp amplifier (HEKA-Electronik). The signals were filtered at 3 kHz and digitized at 20 kHz. On-line data acquisition and off-line data analysis were performed using PULSE software (HEKA-Electronik). Stimulation pipettes were filled with the standard saline.

CFs were stimulated in the GL at 20–100 μm away from the PC soma through a patch pipette filled with the normal external solution with stimuli (duration, 0.1 ms; amplitude, 0–100 μA) repeated at 0.2 Hz. When a CF was stimulated, EPSCs were elicited in an all-or-none manner in the majority of control PCs, indicating that such PCs were innervated by single CFs. CF-mediated EPSCs (CF-EPSCs) displayed prominent paired-pulse depression with inter-stimulus interval of 50–100 ms, which enabled us to distinguish them from PF-EPECs that displayed clear paired-pulse facilitation^64^. In some PCs, more than one discrete CF-EPSCs could be elicited when the stimulus intensity was increased gradually as exemplified in Supplementary Fig. 4. The number of CFs innervating the PC was estimated by counting the number of discrete CF-EPSC steps. To confirm the number of CFs innervating PC under recording, the stimulation electrode was moved systematically around the PC soma within the GL. Amplitude of CF-EPSCs was measured at the holding potential of −10 mV. The holding potential was corrected for liquid junction potential. For the CF-EPSCs whose amplitudes were smaller than 100 pA, they were evoked again at −70 mV to confirm that they represented discrete EPSC steps and paired-pulse depression. To examine the effects of knocking down BDNF in PCs, PCs were sampled at locations in which virus-infected PCs were rich (mOrange2-positive regions) and from locations in which transfected PCs were absent (mOrange2-negative regions). CF innervation patterns were compared between mOrange2-positive (BDNF KD) and mOrange2-negative (Control) regions in the same slices. To examine the effects of knocking down TrkB or p75^NTR^ in CFs, PCs were sample at locations in which virus-infected CFs were rich (EGFP-positive regions) (Fig. 5c) and from locations in which transfected CFs were absent (EGFP-negative regions). CF innervation patterns were compared between EGFP-positive (TrkB KD/p75^NTR^ KD) and EGFP-negative (Control) regions in the same slices. PFs were stimulated in the ML, and PF-EPSCs were recorded at the holding potential of −70 mV with gradually decreasing stimulus intensity from 10 to 1 μA.

### Quantification of disparity in multiple CF-EPSCs

To quantify the disparity in multiple CF-EPSCs recorded in a given PC, we calculated two parameters, disparity ratio, and disparity index^11^.

For calculating disparity ratio, the amplitude of individual CF-EPSCs in a given multiply innervated PC were measured and they were numbered in the order of their amplitudes (*A*_1_, *A*_2_, … , *A*_*N*_, *N* ≧ 2, *A*_*N*_ represents the largest CF-EPSC). The disparity ratio was obtained from the following formula.

$${\rm{Disparity}}\,{\rm{ratio = }}\left( {{A_{\rm{1}}}{\rm{/}}{A_N}{\rm{ + }}{A_{\rm{2}}}{\rm{/}}{A_N}{\rm{ + \ldots + }}{A_{N - {\rm{1}}}}{\rm{/}}{A_N}} \right){\rm{/}}\left( {N - 1} \right)$$

If all the CF-EPSCs in a given PC have the same amplitudes, the disparity ratio will be 1. Conversely, if the differences between *A*_*N*_ and other smaller CF-EPSCs are large, the disparity ratio will be small.

The disparity index was obtained from the following formula.

$${\rm{Disparity}}\,{\rm{index = S}}{\rm{.D}}{\rm{./}}M$$

$$M{\rm{ = }}{\sum} {{A_i}{\rm{/}}N\left( {i{\rm{ = 1,2,3,}} \ldots N{\rm{;}}\,N \ge 2} \right)} $$

$${\rm{S}}{\rm{.D}}{\rm{. = }}{\sum} {{{\left( {{A_i}{\rm{-}}M} \right)}^{\rm{2}}}{\rm{/}}\left( {N - 1} \right)} $$

*A*_*i*_ is the CF-EPSC amplitude recorded at the same holding potential, and *N* is the number of CFs in a given PC. The disparity index is the coefficient of variation for all CF-EPSC amplitudes recorded in a given PC. Hence, if the amplitudes of CF-EPSC are identical, the value of the disparity index will be small. Conversely, if CF-EPSCs are variable in size, the value will be large^11^.

### Morphological analysis

Under deep pentobarbital anesthesia (100 μg/g of body weight, i.p.), mice were perfused with 4% paraformaldehyde in 0.1 M phosphate buffer, and processed for preparation of parasagittal microslicer sections (50 μm in thickness). After permeabilization and blocking of nonspecific binding of antibodies, cerebellar sections were incubated with the following primary antibodies overnight (1 μg/ml): the rat DsRed polyclonal antibody against mOrange2 fluorescent protein (#632496, 1:1000, Clontech) to visualize virus-infected PCs, the guinea pig antibody against calbindin D28K (Calbindin-GP-Af280, 1:200, Frontier Institute) to label PCs, the rabbit antibody against VGluT2 (VGluT2-Rb-AF720, 1:200, Frontier Institute) to label CF terminals and the rabbit anti-TrkB antibody which was raised against the 366–432 amino acid residues (NM_001025074) by T. Miyazaki and M. Watanabe. After incubation with a mixture of species-specific secondary antibodies (anti-rat Alexa Fluor488 antibody, 1:300, Jackson immunoresearch; anti-mouse Cy5 antibody, 1:300, Jackson immunoresearch; anti-rabbit Cy3 antibody, 1:300, Jackson) for 2 h, the immunolabeled sections were washed and then examined with a confocal laser scanning microscope (FV1200, Olympus).

### Statistical analysis

All data are presented as mean ± standard error of the mean (s.e.m.). Error bars in the figures also indicate s.e.m. (shown only if larger than symbol size).

No statistical method was used to predetermine sample sizes, but sample sizes are similar to previous publications^26, 27, 39^. No samples or animals were excluded from the analyses.

The normality of the samples was assessed with a Shapiro–Wilk test. Statistical significance between two independent samples was assessed by Student’s *t* test or Mann–Whitney *U*-test for not normally distributed data. To compare the two different categorical independent samples on one dependent variable, two-way repeated measured ANOVA with Tukey’s post-hoc test was used as indicated in the text. Comparisons between more than two groups were analyzed using the Kruskal–Wallis test followed by Dunn’s multiple comparisons test. Equality of variance for unpaired *t*-test and two-way ANOVA was verified using *F*-test and Levene’s test, respectively. All tests used in this paper were two-sided.

For the experiments on knockout mice and their littermate controls, data were obtained in a blind manner in which the experimenters did not know the mouse genotypes. For the experiments on PCs with lentivirus-mediated knockdown of molecules, data were obtained in a non-blinded manner.

Randomization was not performed.

Statistical analyses and plotting graphs were performed with GraphPad Prism 5 (GraphPad software) and IBM SPSS Statistics 24 (IBM). Differences between groups were judged to be significant when *P* values were smaller than 0.05. *, **, ***, **** and ns represents *P* < 0.05, *P* < 0.01, *P* < 0.001, *P* < 0.0001 and not significant, respectively.

### Data availability

The data that support the findings of the present study are available within the paper and its Supplementary Information files, or available from the corresponding author upon reasonable request.

## Electronic supplementary material

Supplementary Information
