# Supplementary material for: Retrograde BDNF to TrkB signaling promotes synapse elimination in the developing cerebellum
Source: Nat Commun. 2017 Aug 4;8:195. doi: 10.1038/s41467-017-00260-w (PMC5543168; doi:10.1038/s41467-017-00260-w)
Supplement: Supplementary file 1 — Supplementary Information [file 41467_2017_260_MOESM1_ESM.pdf]

File name: Supplementary Information
Description: Supplementary Figures and Supplementary Tables

Supplementary Information

Supplementary figures

Supplementary Fig. 1

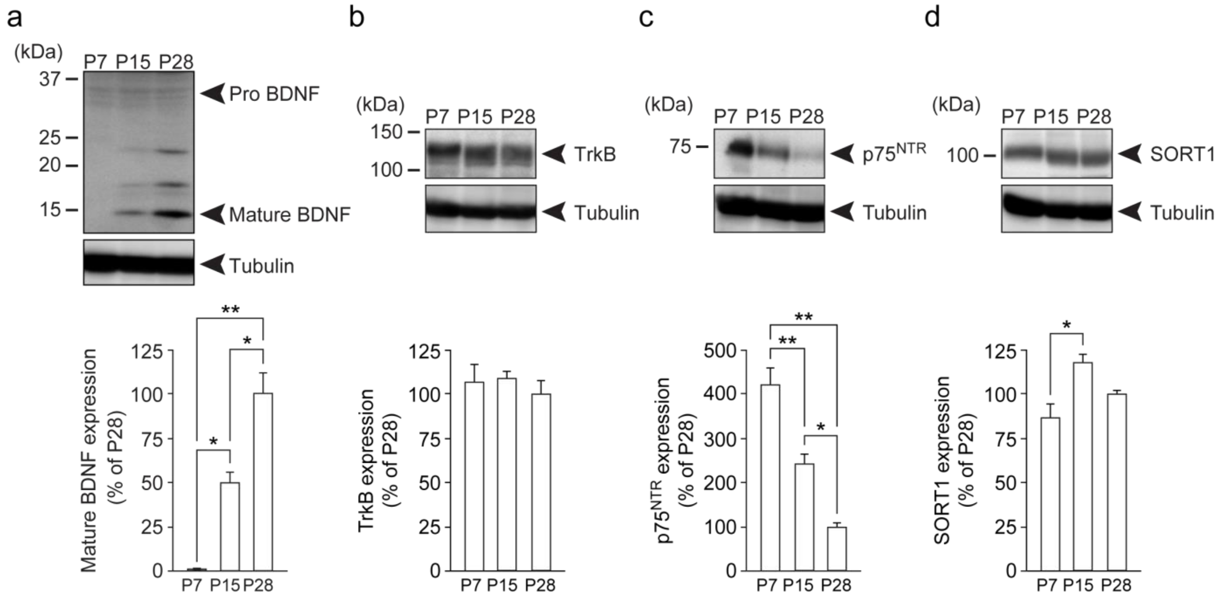

Supplementary Fig. 1. Expression of BDNF and its putative receptors in the developing cerebellum.

(**a**) (Upper panel) Sample Western blot data for pro BDNF and mature BDNF in the cerebellum of control mice at P7, P15 and P28. (lower panel) Summary bar graph showing the level of mature BDNF expression in the wild-type cerebellum of the corresponding ages (one-way ANOVA, n = 3 mice each, P7: 0.4 ± 0.4, P15: 49.8 ± 5.6, P28: 100.0 ± 12.7, P7 versus P15, P = 0.014; P7 versus P28, P = 0.0004; P15 versus P28, P = 0.013). Proteins loaded in each lane are 30 μg. Protein levels were normalized to the averaged value of the BDNF level at P28. Error bars indicate SEM. *P < 0.05, **P < 0.01.

(**b-d**) Similar to (**a,b**) but for results showing the expression of TrkB (**b**) (one-way ANOVA, n = 3 mice each, P7: 106.2 ± 10.2, P15: 109.1 ± 4.6, P28: 100.0 ± 8.0), p75^{NTR} (**c**) (one-way ANOVA, n = 3 mice each, P7: 420.5 ± 37.6, P15: 241.7 ± 22.3, P28: 100.0 ± 7.5, P7 versus P15, P = 0.0078; P7 versus P28, P = 0.0003; P15 versus P28, P = 0.024) and SORT1 (**d**) (one-way ANOVA, n = 3 mice each, P7: 85.1 ± 7.2, P15: 116.6 ± 4.6, P28: 100.0 ± 1.3, P7 versus P15, P = 0.013). Error bars indicate SEM. *P < 0.05, **P < 0.01. ns indicates no significant difference between the two groups. The uncropped images for the western blot data are shown in Supplementary Fig. 14**a**.

Supplementary Fig. 2

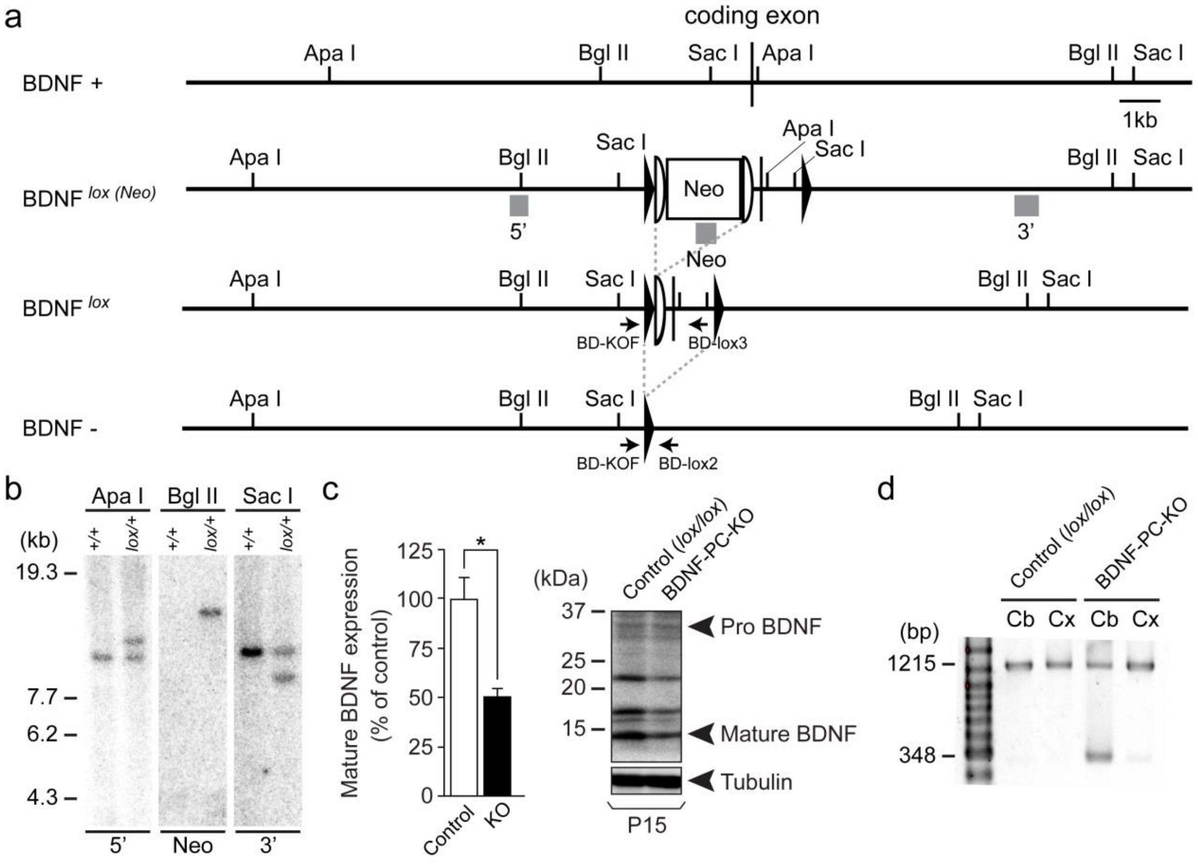

Supplementary Fig. 2. Generation of PC-specific BDNF knockout (BDNF-PC-KO) mice

(**a**) Schematic representations of *Bdnf* genomic DNA (BDNF +), targeted genome (BDNF $^{lox\ (Neo)}$), targeted genome after FLP-mediated recombination (BDNF ^{lox}) and Cre-mediated deleted genome (BDNF -). Solid triangles indicate *loxP* sequences. Open semicircles indicate flippase recognition target (FRT) sequences. Shaded bars indicate the probes (5', Neo, 3') for Southern blot analysis.

(**b**) Southern blot analysis of genomic DNA prepared from wild-type (+/+) and BDNF *lox/+* (*lox/+*) ES cells. (Left) Apa I - digested DNA hybridized with a 5' probe: 10.5 kb for wild-type and 12.1 kb for floxed allele. (Middle) Bgl II - digested DNA hybridized with a Neo probe: 14.5 kb for floxed allele. (Right) Sac I - digested DNA hybridized with 3' probe: 10.2 kb for wild-type and 8.3 kb for floxed allele.

(**c**) Western blot analysis of the homogenates from the cerebellum of Control and BDNF-PC-KO mice at P15. (left) Bar graph showing the mature BDNF expression level of control (*lox/lox*) and BDNF-PC-KO mice (control: 100±11.14, BDNF-PC-KO: 50.47 ± 4.20, n = 3 mice each, P = 0.0141, Student's t test). (right) Sample Western blot data for pro BDNF and mature BDNF in the cerebellum of Control and BDNF-PC-KO mice at P15. Proteins loaded in each lane are 30 μg. Protein levels

were normalized to tubulin levels. Error bars indicate SEM. *$P < 0.05$. The uncropped images for the western blot data are shown in Supplementary Fig. 14**b**.
(**d**) PCR analysis of genomic DNA prepared from the cerebellum and cerebral cortex of Control and BDNF-PC-KO mice. DNA loaded in each lane is 50 ng. The primers used in the PCR are indicated in (**a**). For primer sequences see **Methods**.

Supplementary Fig. 3

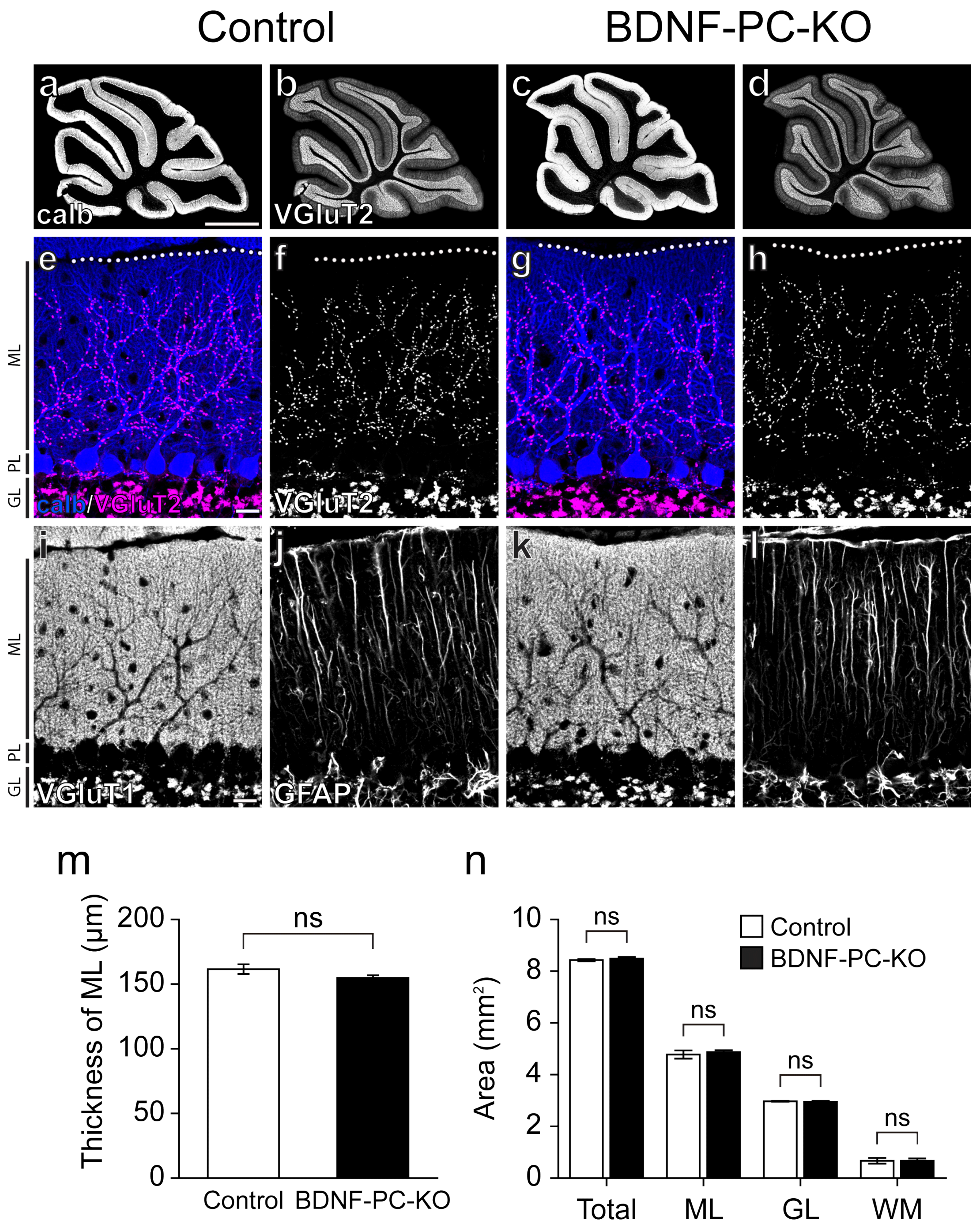

Supplementary Fig. 3. Morphology of the cerebellum and its cellular elements of BDNF-PC-KO mice

(**a-d**) Immunofluorescence for calbindin (calb) (**a,c**) and VGluT2 (**b,d**) in the cerebellum of a littermate control (**a,c**) and a BDNF-PC-KO (**b,d**) mouse.
(**e-h**) Cerebellar sections of a littermate control (**e**) and a BDNF-PC-KO (**g**) mouse double-immunostained for calbindin (blue) and VGluT2 (magenta). **f** and **h** are the same images of **e** and **g**, respectively, but only the signals for VGluT2 are shown. Dotted lines in **e-h** indicate the pial surface.
(**i-l**) Cerebellar sections of a littermate control (**i,j**) and a BDNF-PC-KO (**k,l**) mouse immunostained for VGluT1 (**i,k**). and GFAP (**j,l**).
(**m**) Summary bar graph showing the thickness of the molecular layer (ML) of the cerebellar cortex for control (n = 21) and BDNF-PC-KO (n = 22) mice (n = 3 mice each, control: 161.6 ± 3.8, BDNF-PC-KO: 154.8 ± 2.1, *P* = 0.2153, Mann-Whitney *U*-test). ns indicates no significant difference between the two groups.
(**n**) Summary bar graph showing the area of the cerebellar cortex for control and BDNF-PC-KO mice. Areas of the total cerebellar cortex (control: 8.42 ± 0.04, BDNF-PC-KO: 8.48 ± 0.07, *P* = 0.5516, Mann-Whitney *U*-test), the ML (control: 4.78 ± 0.16, BDNF-PC-KO: 4.87 ± 0.07, *P* = 0.6282, Mann-Whitney *U*-test), the granule cell layer (GL) (control: 2.97 ± 0.02, BDNF-PC-KO: 2.94 ± 0.04, *P* = 5305, Mann-Whitney *U*-test) and the white matter (WM) (control: 0.67 ± 0.11, BDNF-PC-KO: 0.67 ± 0.09, *P* = 0.9792, Mann-Whitney *U*-test) were measured from 3 mice each. ns indicates no significant difference between the two groups.
Layers in the cerebellar cortex are indicated beside e and i: ML, molecular layer; PL, Purkinje cell layer; GL, granule cell layer.
Scale bars: **a-d**, 1 mm; **e-l**, 20 μm

Supplementary Fig. 4

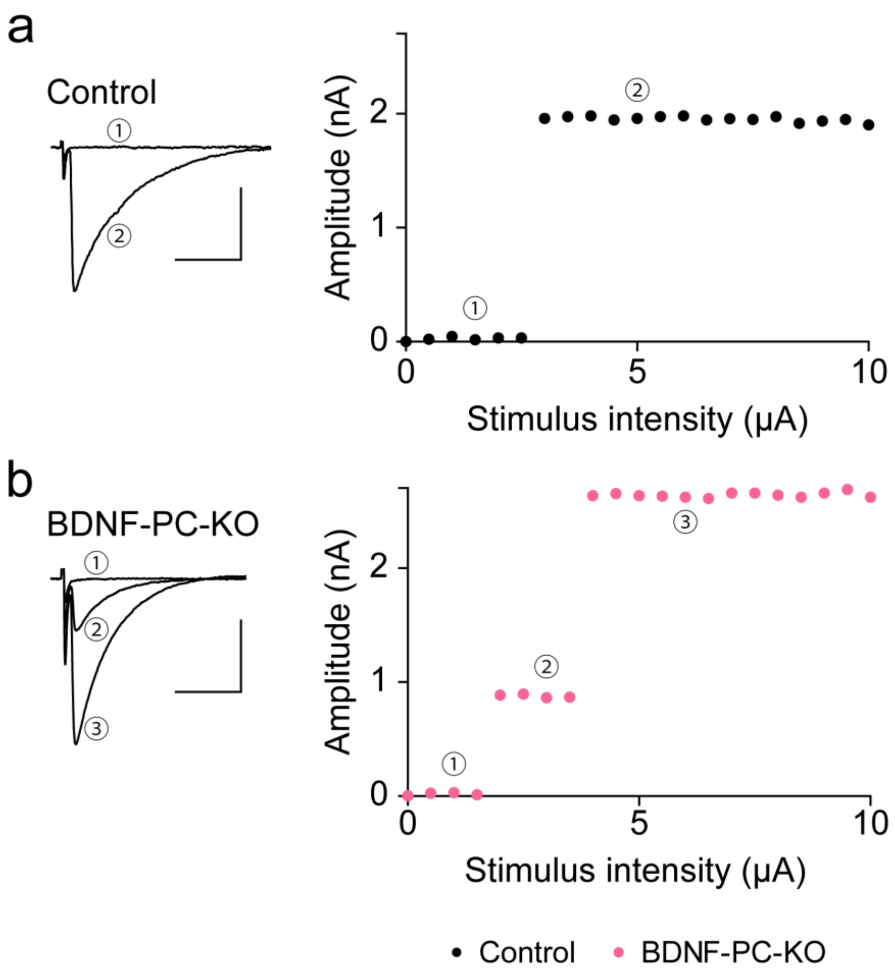

Supplementary Fig. 4. Examples of CF-EPSCs in response to increasing stimulus intensity in wild-type and BDNF-PC-KO mice.

(**a**) Sample traces (left) and input-output relationship of CF-EPSCs in a control PC (P31). Single traces at CF stimulus intensity of 1.5 μA (1) and 5 μA (2) were superimposed. Note that CF-EPSCs were elicited in an all-or-none fashion at the threshold of 3 μA. This PC was judged to be innervated by a single CF.

(**b**) Similar to (**a**) but from a BDNF-PC-KO PC (P30). Single traces at CF stimulus intensity of 1 μA (1), 3 μA (2) and 6 μA (3) were superimposed. Note that CF-EPSCs were elicited in a stepwise manner at 2 μA and at 4 μA. This PC was judged to be innervated by two CFs.

Holding potential was -10 mV. Scale bars, 10 ms and 1 nA.

Supplementary Fig. 5

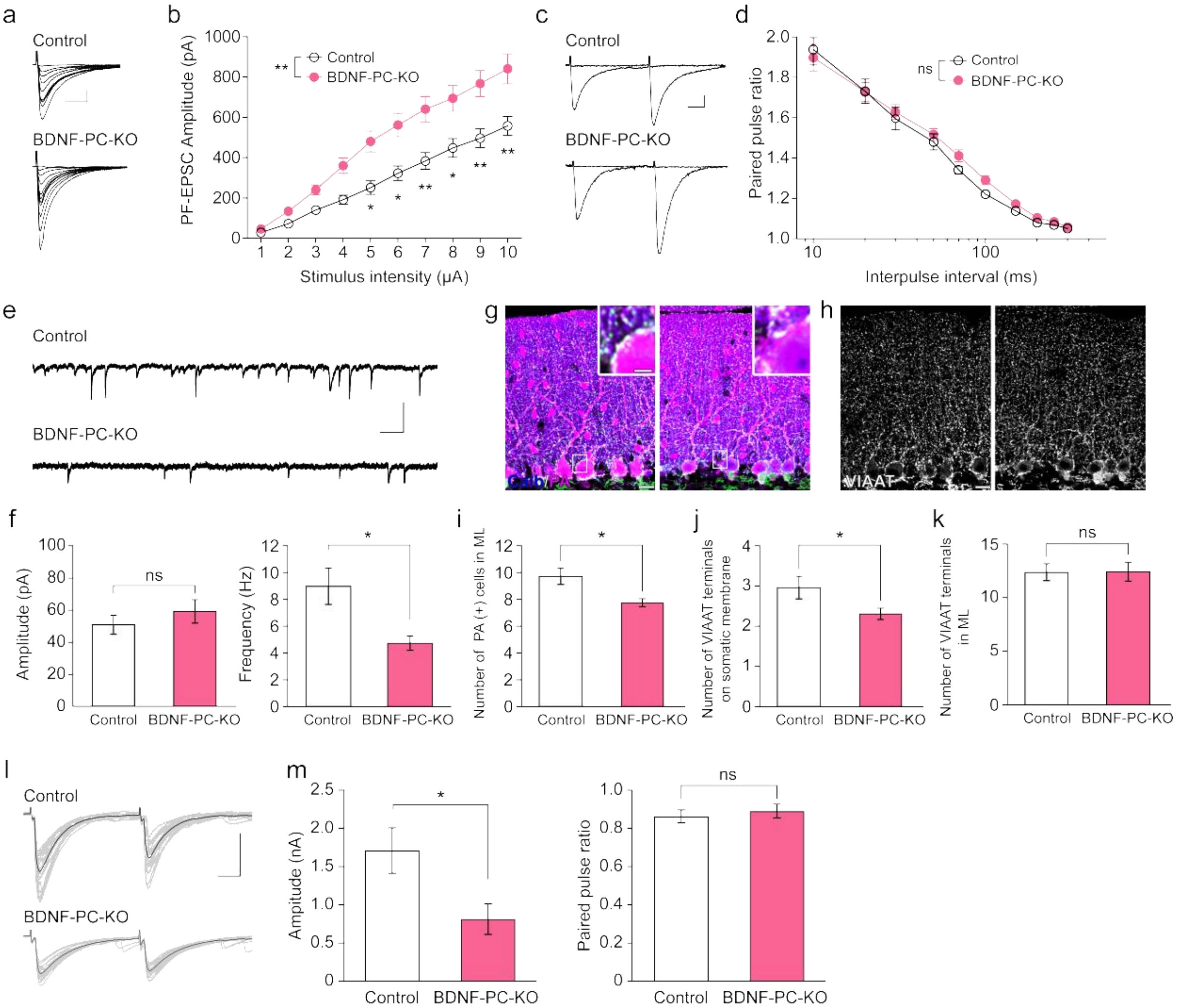

Supplementary Fig. 5. Enhanced PF-mediated excitation and reduced inhibition to PCs of mature BDNF-PC-KO mice.

(**a,b**) Representative traces of PF-EPSC (**a**) and input-output relationship of PF-EPSCs (**b**) with increasing stimulus intensities by 1 μA step from 1 to 10 μA for control (n = 14 PCs from 4 mice) and BDNF-PC-KO (n = 14 PCs from 4 mice) mice during P21 to P35 (Repeated-measures two-way ANOVA with Tukey's post hoc test: factor genotype, F(1,234) = 13.11, *P* = 0.0012; factor stimulus, F(9,234) = 170.1, *P* < 0.0001; interaction, F(9,234) = 7.47, *P* < 0.0001; and Tukey's post hoc test, 5 μA, *P* = 0.032; 6 μA, *P* = 0.017; 7 μA, *P* = 0.006; 8 μA, *P* = 0.012; 9 μA, *P* = 0.0024; 10 μA, *P* = 0.001). PF-EPSCs in (**a**) were recorded from a control (top, P21) and a BDNF-PC-KO (bottom, P21) mouse. Holding potential was -70 mV. Scale bars in (**a**), 10 ms and 100 pA. In (**b**), each point represents mean ± SEM. *$P < 0.05$, **$P < 0.01$.

(**c,d**) Representative traces of PF-EPSCs to paired stimuli with inter-pulse interval of 50 ms (**c**) and summary graph (**d**) showing averaged values of paired-pulse ratio in wild-type (open circles; n = 17 PCs from 8 mice) and BDNF-PC-KO (filled circles; n = 17 PCs from 9 mice) mice during P21 to P35 (Repeated-measures two-way ANOVA

with Tukey's post hoc test: factor genotype, $F(1,288) = 0.511$, $P = 0.479$; factor stimulus, $F(9,288) = 215.1$, $P < 0.0001$; interaction, $F(9,288) = 0.635$, $P = 0.767$). PF-EPSCs in (**c**) were recorded from a wild-type (P21; top) and a BDNF-PC-KO (P21; bottom) PC. Stimulus pairs were applied at 0.2 Hz. Holding potential was -70 mV. Scale bars in (**c**), 10 ms and 100 pA. In (**d**), each point represents mean ± SEM. ns indicates no significant difference.

(**e,f**) Sample traces of mIPSCs (**e**) and summary bar graphs (**f**) showing the average amplitude (left, control: 50.87 pA ± 7.28, BDNF-PC-KO: 59.20 pA ± 7.28, $P = 0.450$, Mann-Whitney *U*-test) and frequency (right, control: 8.98 Hz ± 1.36, BDNF-PC-KO: 4.73 Hz ± 0.52, $P = 0.029$, Mann-Whitney *U*-test) of mIPSCs from PCs at P15 - 16 of wild-type (n = 15 PCs from 4 mice) and BDNF-PC-KO (n = 17 PCs from 4 mice) mice. mIPSCs were recorded at -70 mV with symmetrical Cl^- concentrations in the presence of 10 μM NBQX, 5 μM R-CPP, and 1 μM TTX in the extracellular solution. Scale bars in (**e**), 10 ms and 100 pA. In (**f**), each point represents mean ± SEM. *$P < 0.05$; ns indicates no significant difference between the two groups.

(**g,h**) Cerebellar sections of a littermate control (**g, h, left panels**) and a BDNF-PC-KO (**g, h, right panels**) mouse double immunostained with anti-calbindin antibody (blue) and anti-parvalbumin antibody (magenta) (**g**) or immunostained with anti-VIAAT antibody (green) (**h**). The insets in **g** are enlarged images of the boxed areas.

(**i-k**) Bar graphs showing the number of parvalbumin-positive cells within 10,000 μm2 of the molecular layer area (control: 9.69 ± 0.58, BDNF-PC-KO: 7.98 ± 0.30, $P = 0.031$, Mann-Whitney *U*-test) (**i**), the number of VIAAT-positive terminals on PC somata per 10 μm along somatic plasma membrane (control: 2.95 ± 0.28, BDNF-PC-KO: 2.92 ± 0.14, $P = 0.029$, Mann-Whitney *U*-test) (**j**), and the number of VIAAT-positive terminals within 100 μm^2 of the molecular layer area (control: 12.26 ± 0.77, BDNF-PC-KO: 12.31 ± 0.88, $P = 0.965$, Mann-Whitney *U*-test) (**k**), for wild-type (open columns) and BDNF-PC-KO (filled columns) mice. Each point and error bar represents mean ± SEM. *$P < 0.05$; ns indicates no significant difference between the two groups.

(**l,m**) Sample traces of eIPSCs (**l**) and summary bar graphs (**m**) showing the average amplitude (left, control: 1.71 pA ± 0.30, BDNF-PC-KO: 0.81 pA ± 0.12, $P = 0.012$, Mann-Whitney *U*-test) and paired-pulse ratio (right, inter-stimulus interval of 50 ms, control: 0.86 ± 0.03, BDNF-PC-KO: 0.89 ± 0.04, $P = 0.496$, Mann-Whitney *U*-test) of eIPSCs from PCs of control mice (n = 12 PCs from 3 mice) and BDNF-PC-KO mice (n = 13 PCs from 3 mice) at P15. eIPSCs were recorded at -70 mV with symmetrical Cl^- concentrations in the presence of 10 μM NBQX, 5 μM R-CPP, and 1 μM TTX in the extracellular solution. Scale bars in (**l**), 10 ms and 1 nA. Each point and error bar represents mean ± SEM. *$P < 0.05$; ns indicates no significant difference between the two groups.

Supplementary Fig. 6

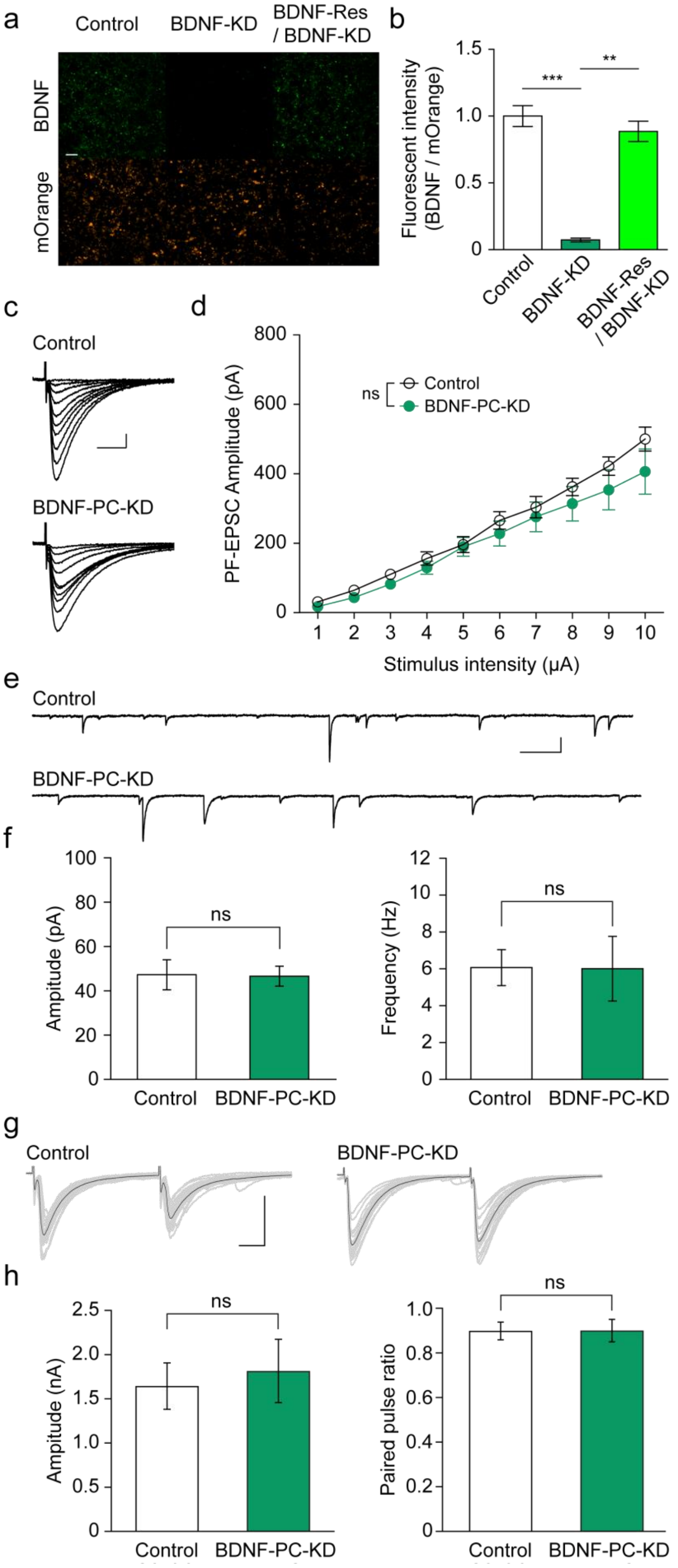

Supplementary Fig. 6. PF EPSCs and mIPSCs are not altered in PCs with BDNF-KD by microRNA vector.

(**a**) Efficacy of BDNF knockdown by microRNA vector. Double immunostaining with anti-DsRed (shown in orange signal) and anti-BDNF antibodies to detect BDNF (shown in green signal) in HEK 293T cells transfected simultaneously with BDNF-expression vectors and an mOrange-expression vectors (left, Control), with BDNF-expression vectors together with a microRNA vector against BDNF tagged with mOrange fluorescence protein (middle, BDNF-KD), or with an RNA interference (RNAi)-resistant BDNF vector together with a microRNA vector against BDNF tagged with mOrange fluorescence protein (right, BDNF-Res). Scale bar, 30 µm.

(**b**) Bar graph showing fluorescence intensity of BDNF relative to that of mOrange in control (1.00 ± 0.08, n = 10 areas), BDNF-KD (0.07 ± 0.01, n = 10 areas) and BDNF-Res (0.89 ± 0.07, n = 10 areas) cells ($P < 0.0001$, Kruskall-Wallis test with Dunn's multiple comparison post hoc test). **$P < 0.01$, ***$P < 0.001$.

(**c,d**) Representative traces of PF-EPSC (**c**) and Input-output relationship of PF-EPSCs (**d**) with increasing stimulus intensities by 1 µA step from 1 to 10 µA for control (open circles, n = 8 PCs from 4 mice) and BDNF-KD (filled circles, n = 8 PCs from 4 mice) PCs recorded in mice during P21 to P30 (Repeated-measures two-way ANOVA with Tukey's post hoc test: factor gene KD, $F(1,126) = 1.16$, $P = 0.2996$; factor stimulus, $F(9,126) = 97.99$, $P < 0.0001$; interaction, $F(9,126) = 0.8207$, $P = 0.5982$). PF-EPSCs in (**c**) were recorded from a control (top, P21) and a BDNF-PC-KD (bottom, P21) mouse. Holding potential was -70 mV. Scale bars in (**c**), 10 ms and 100 pA. In (**d**), each point represents mean ± SEM. ns indicates no significant difference between the two groups.

(**e,f**) Sample traces of mIPSCs (**e**) and summary bar graphs showing the average amplitude (left, control: 47.27 pA ± 6.78, BDNF-PC-KD: 46.65 pA ± 4.50, $P = 0.7430$, Mann-Whitney *U*-test) and frequency (right, control: 6.07 pA ± 7.28, BDNF-PC-KO: 6.01 pA ± 1.75, $P = 0.6058$, Mann-Whitney *U*-test) of mIPSCs (**f**) of in control (n = 8 PCs from 3 mice) and BDNF KD (n = 9 PCs from 3 mice) PCs at P15-16. mIPSCs were recorded similarly to Supplementary Fig. 5**e**. ns indicates no significant difference between the two groups.

(**g,h**) Sample traces of eIPSCs (**g**) and summary bar graphs (**h**) showing the average amplitude (left, control: 1.65 pA ± 0.26, BDNF-PC-KD: 1.82 pA ± 0.36, $P = 0.885$, Mann-Whitney *U*-test) and paired-pulse ratio (right, with inter-stimulus interval of 50 ms, control: 0.90 ± 0.04, BDNF-PC-KD: 0.90 ± 0.05, $P = 0.773$, Mann-Whitney *U*-test) of eIPSCs from PCs of control mice (n = 12 PCs from 3 mice) and BDNF-PC-KD mice (n = 12 PCs from 3 mice) at P15. eIPSCs were recorded similarly to Supplementary Fig. 5**l** . Scale bars in (**g**), 10 ms and 1 nA. Each point and error bar represents mean ± SEM. ns indicates no significant difference between the two groups.

Supplementary Fig. 7

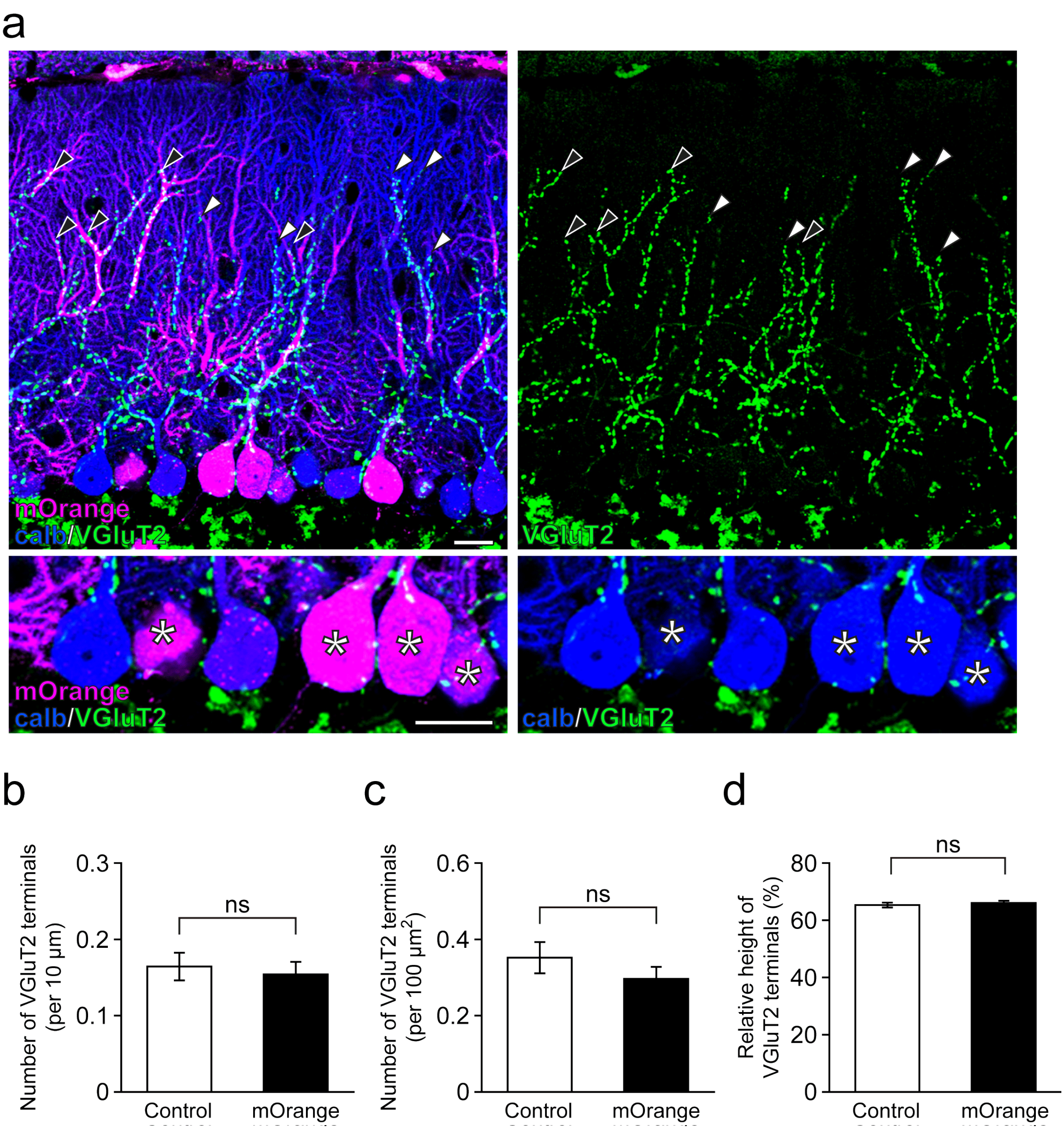

Supplementary Fig. 7. Expression of mOrange alone in PCs does not affect the morphology of CF innervation.

(**a**) Triple fluorescent labeling for calbindin (blue), VGluT2 (green), and mOrange positive PCs (magenta) from a P30 mouse. Somata of PCs in the upper left panel are shown enlarged in the lower panels. Asterisks indicate the somata of mOrange positive PCs. White and black arrowheads indicate the most distal tip of VGluT2-positive CF terminals along dendrites of control and mOrange positive PCs, respectively.

(**b, c**) Summary bar graphs showing the number of VGluT2-positive terminals per 10 μm membrane of the PC soma **(b)** and the number of VGluT2-positive terminals per 100 μm^2 of the PC somatic area **(c)**. Data were collected from mOrange-nagative control PCs (n = 111) and mOrange-positive PCs (n = 95) from 3 mice (for **b**, control PC: 0.16 ± 0.02, mOrange: 0.15 ± 0.02, *P* = 0.6961; for **c**, control PC: 0.35 ± 0.04, mOrange: 0.30 ± 0.03, *P* = 0.6156, Mann-Whitney *U*-test).
(**d**) Summary bar graph showing the relative height of CF terminals to the thickness of the ML for mOrange-negative control PCs (n = 116) and mOrange-positive PCs (n = 106), from 3 mice (control PC: 65.5 ± 0.82, mOrange: 66.2 ± 0.81, *P* = 0.7284, Mann-Whitney *U*-test). The distance between the most distal tip of VGluT2-posotive CF terminals along dendrites and the center of the soma was measured for each PC and the value was divided by the thickness of the ML. Each point and error bar represent mean ± SEM. ns indicates no significant difference between the two groups.
Scale bars: 20 μm

Supplementary Fig. 8

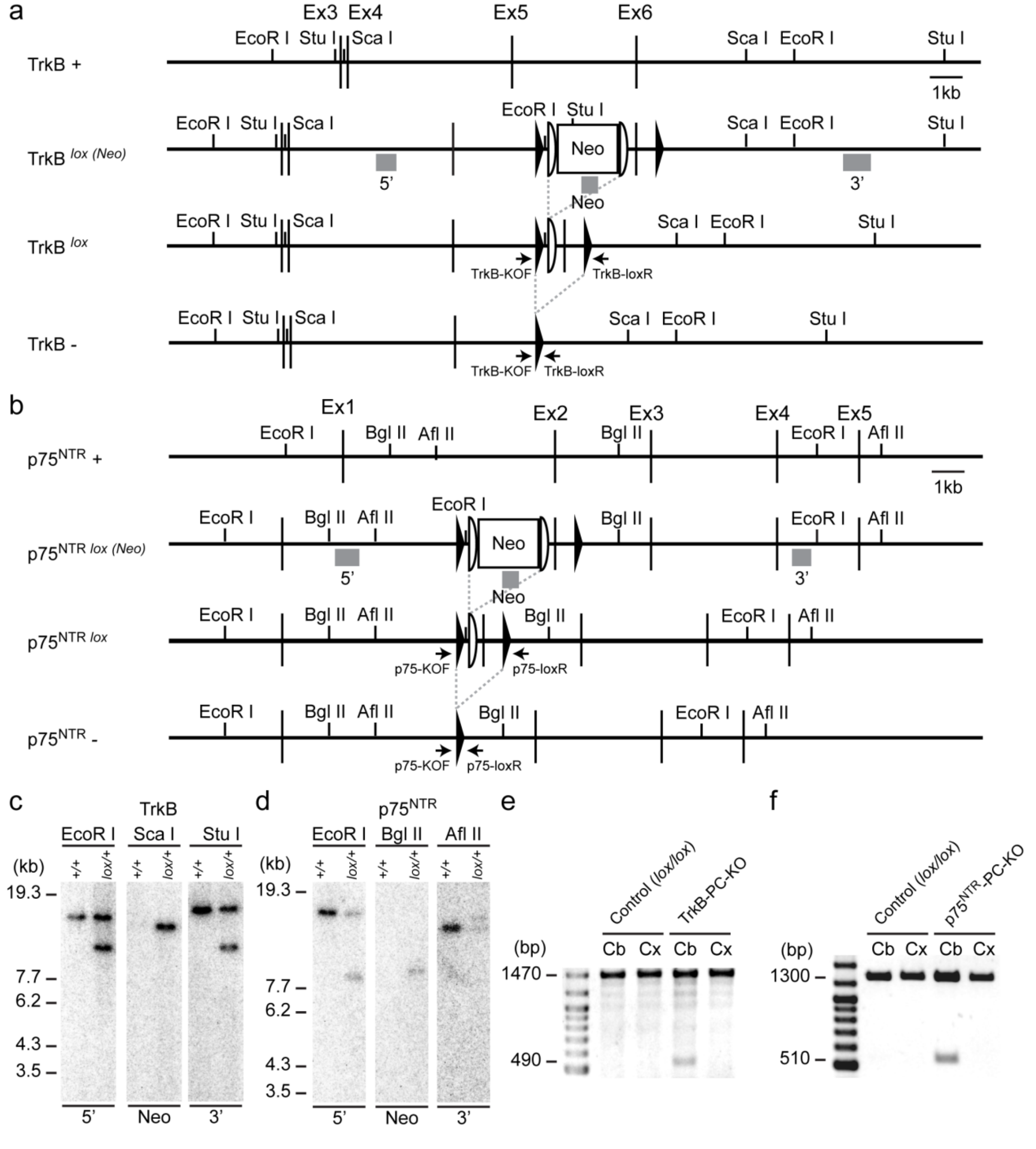

Supplementary Fig. 8. Generation of PC-specific TrkB knockout (TrkB-PC-KO) mice and PC-specific p75^{NTR}-knockout (p75^{NTR}-PC-KO) mice.

(**a**) Schematic representations of TrkB genomic DNA (TrkB +), targeted genome (TrkB $^{lox(Neo)}$), targeted genome after FLP-mediated recombination (TrkB ^{lox}) and Cre-mediated deleted genome (TrkB -). Solid triangles indicate *loxP* sequences. Open semicircles indicate FRT sequences. Shaded bars indicate the probes (5', Neo, 3') for Southern blot analysis.

(**b**) Schematic representations of p75^{NTR} genomic DNA (p75^{NTR} +), targeted genome

($p75^{NTR\ lox\ (Neo)}$), targeted genome after FLP-mediated recombination ($p75^{NTR\ lox}$) and Cre-mediated deleted genome ($p75^{NTR}$ -). Solid triangles indicate *loxP* sequences. Shaded bars indicate the probes (5', Neo, 3') for Southern blot analysis.

(**c**) Southern blot analysis of genomic DNA prepared from wild-type (+/+) and TrkB *lox*/+ (*lox*/+) ES cells.

(Left) EcoR I - digested DNA hybridized with a 5' probe: 15.7 kb for wild-type and 10.6 kb for floxed allele.

(Middle) Sca I - digested DNA hybridized with a Neo probe: 13.9 kb for floxed allele.

(Right) Stu I - digested DNA hybridized with 3' probe: 19.2 kb for wild-type and 11 kb for floxed allele.

(**d**) Southern blot analysis of genomic DNA prepared from wild-type (+/+) and $p75^{NTR}$ *lox*/+ (*lox*/+) ES cells.

(Left) EcoR I - digested DNA hybridized with a 5' probe: 16 kb for wild-type and 7.9 kb for floxed allele.

(Middle) Bgl II - digested DNA hybridized with a Neo probe: 8.7 kb for floxed allele.

(Right) Afl II - digested DNA hybridized with 3' probe: 13.2 kb for wild-type and 15 kb for floxed allele.

(**e,f**) PCR analysis of genomic DNA prepared from the cerebellum and cerebral cortex of control (*lox/lox*) and TrkB-PC-KO (**e**) or $p75^{NTR}$ -PC-KO (**f**). DNA loaded in each lane is 50 ng. The primers used in the PCR are indicated in (**a**) and (**b**). For primer sequences see **Methods**.

Supplementary Fig. 9

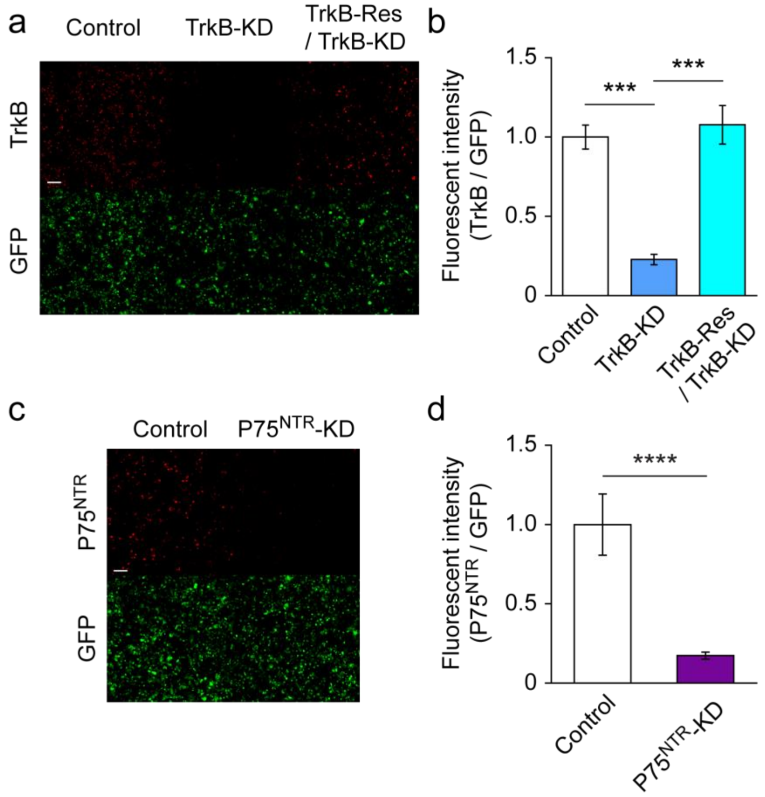

Supplementary figure 9. Efficacy of TrkB and p75^{NTR} knockdown by microRNA vectors.

(**a**) Double immunostaining with anti-GFP (shown as green signal) and anti-TrkB (shown as red signals) antibodies in HEK 293T cells transfected simultaneously with a vector for TrkB-expression and that for GFP-expression (left, Control), with a vector for TrkB-expression together with a microRNA vector against TrkB tagged with GFP (middle, TrkB-KD), or with an RNA interference (RNAi)-resistant TrkB vector together with a microRNA vector against TrkB tagged with GFP (right, TrkB-Res). Scale bar, 30 μm.

(**b**) Summary bar graph showing the fluorescence intensity of TrkB relative to that of GFP in control (1.00 ± 0.08, n = 10 areas), TrkB-KD (0.23 ± 0.03, n = 10 areas), and TrkB -Res (1.08 ± 0.12, n = 10 areas) cells (P < 0.0001, Kruskall-Wallis test with Dunn's multiple comparison post hoc test). ***P < 0.001.

(**c**) Double immunostaining with anti-GFP (shown as green signals) and anti- p75^{NTR} (shown as red signals) antibodies in HEK 293T cells transfected simultaneously with a vector for p75^{NTR} -expression and that for GFP-expression (left, Control) or with a vector for p75^{NTR} -expression together with a microRNA vector against p75^{NTR} tagged with GFP (right, p75^{NTR} -KD). Scale bar, 30 μm.

(**d**) Summary bar graph showing the fluorescence intensity of p75^{NTR} relative to that of GFP in control (1.00 ± 0.19, n = 10 areas) and p75^{NTR} -KD (0.17 ± 0.02, n = 10 areas) cells (P < 0.0001, Mann-Whitney U-test). ****P < 0.0001.

Supplementary Fig. 10

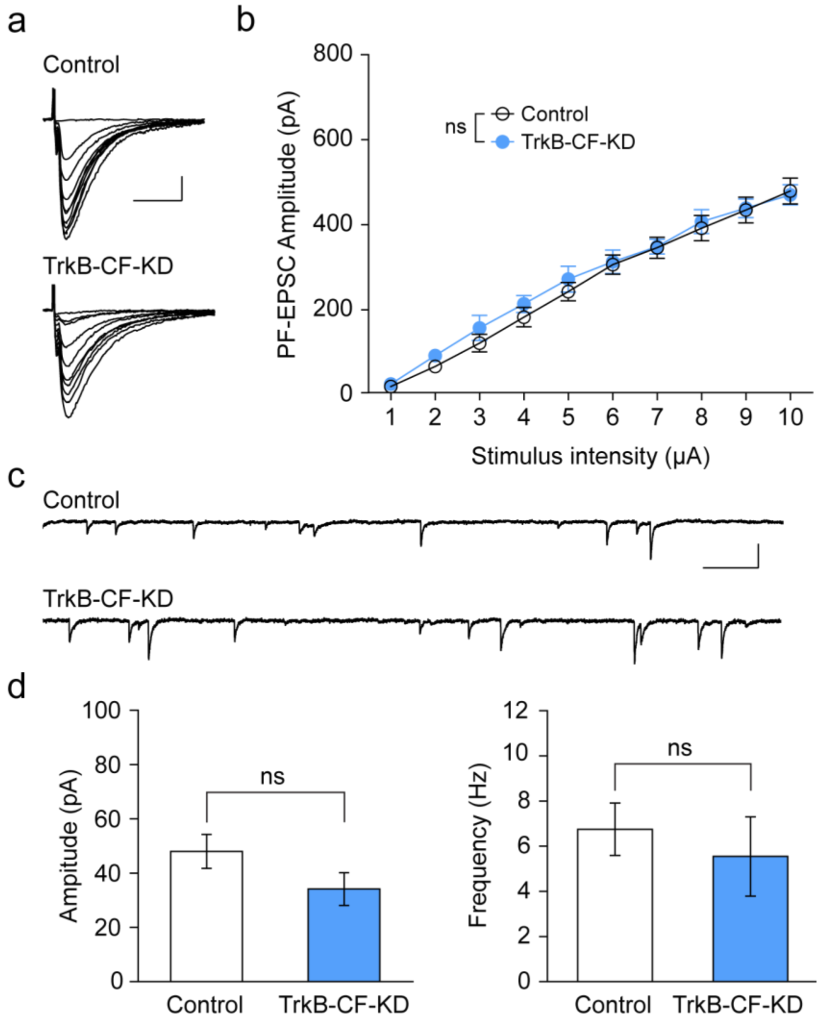

Supplementary Fig. 10. PF-EPSCs and mIPSCs are not altered in PCs associated with TrkB-KD CFs.

(**a,b**) Representative traces of PF-EPSC (**a**) and Input-output relationship of PF-EPSCs (**b**) with increasing stimulus intensities by 1 μA step from 1 to 10 μA for control PCs (open circles, n = 7 PCs from 4 mice) and PCs associated with TrkB-KD CFs (filled circles, n = 7 PCs from 4 mice) recorded in mice during P21 to P30 (Repeated-measures two-way ANOVA with Tukey's post hoc test: factor gene KD, F(1,108) = 0.3061, *P* = 0.5903; factor stimulus, F(9,108) = 236.2, *P* < 0.0001; interaction, F(9,108) = 0.5515, *P* = 0.8336). PF-EPSCs in (**a**) were recorded from a control PC (top, P21) and a PC associated with TrkB-KD CFs (bottom, P21). Holding potential was -70 mV. Scale bars in (**a**), 10 ms and 100 pA. In (**b**), each point and error bar represents mean ± SEM. ns indicates no significant difference between the two groups.

(**c,d**) Sample traces of mIPSCs (**c**) and summary bar graphs showing the average amplitude (left, control: 48.05 pA ± 6.23, BDNF-PC-KO: 34.17 pA ± 6.02, *P* = 0.2677,

Mann-Whitney *U*-test) and frequency (right, control: 6.75 pA ± 1.16, BDNF-PC-KO: 5.55 pA ± 1.76, *P* = 0.6389, Mann-Whitney *U*-test) of mIPSCs (**d**) in control PCs (n = 8 PCs from 3 mice) and PCs associated with TrkB-KD CFs (n = 8 PCs from 3 mice) recorded in mice at P15-16. mIPSCs were recorded similarly to Supplementary Figs. 5**e**. and 6**e**. ns indicates no significant difference between the two groups.

Supplementary Fig. 11

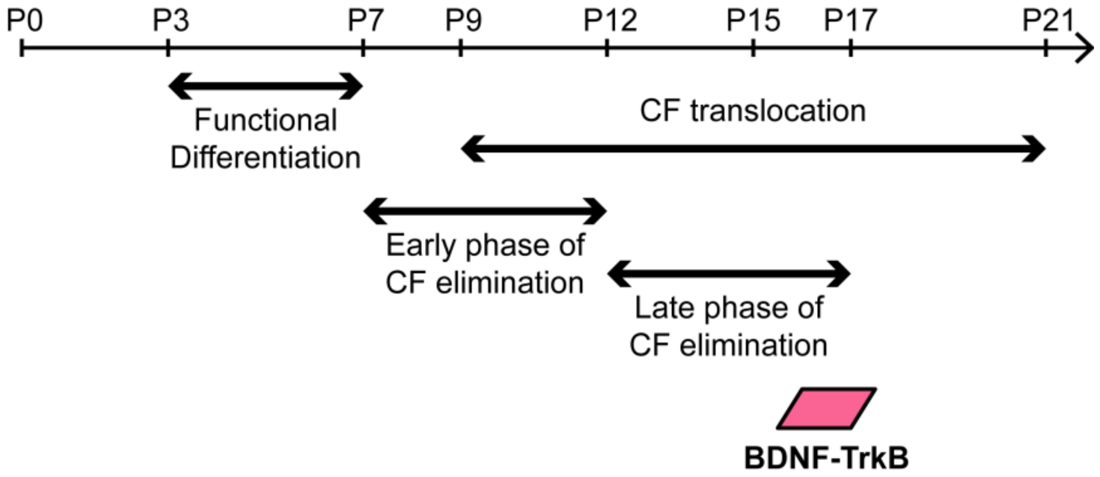

Supplementary Fig. 11. BDNF to TrkB signaling is specifically involved in the final stage of the late phase of CF elimination after P16.

Supplementary Fig. 12

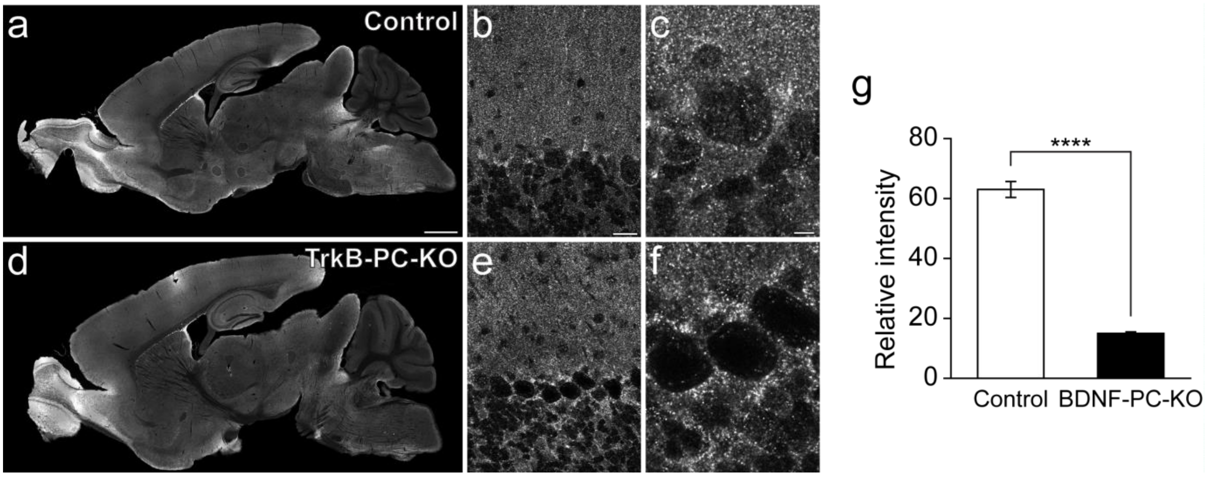

Supplementary Fig. 12. Specificity of anti-TrkB antibody.
(**a-f**) TrkB expression in a control (**a-c**) and a TrkB-PC-KO (**d-f**) mouse. (**b,c**) and (**e,f**) are enlarged images of the cerebellar cortex in (**a**) and (**d**), respectively.
(**g**) Summary bar graph showing the relative intensity of TrkB expression for control and BDNF-PC-KO mice (from 3 mice each, control: 63.04 ± 2.67, BDNF-PC-KO: 15.04 ± 0.45, $P < 0.00001$, Mann-Whitney *U*-test). The relative intensity was calculated as the ratio of signal density in the soma to that in the cerebellar white matter. ****$P < 0.00001$. Scale bars: **a**, 1 mm; **b**, 20 µm; **c**, 5 µm

Supplementary Fig. 13

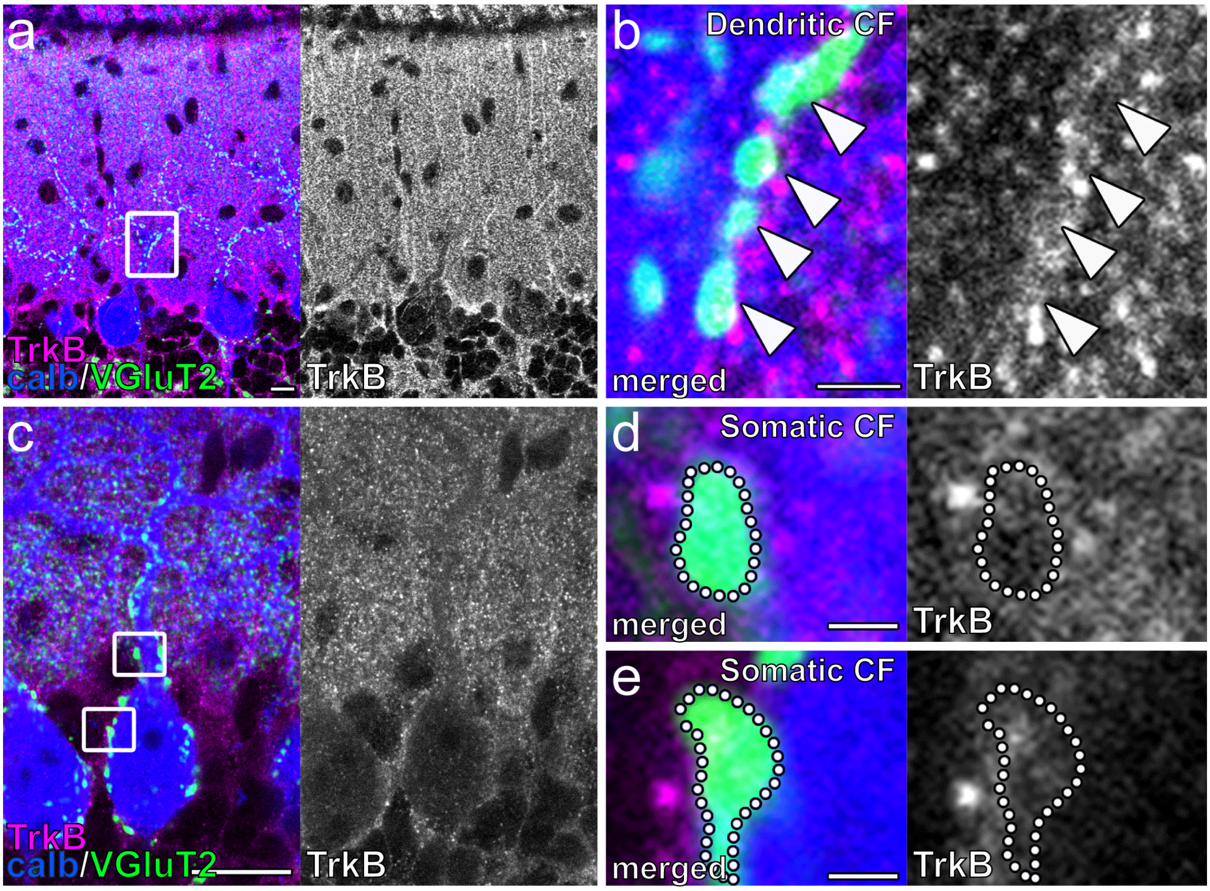

Supplementary Fig. 13. TrkB expression in CFs along dendrites and on somata of PCs.

(**a-d**) Triple fluorescent labeling for calbindin (blue), VGluT2 (green), and TrkB (magenta) in the cerebellum of a P21 mouse. Box in **a** is enlarged in **b**. Upper and lower boxes in **c** are enlarged in **d** and **e**, respectively. White arrowheads in **b** and dotted outline in **d** and **e** indicate the co-localization of TrkB and VGluT2 signals. Scale bars: **a,c** 10 μm; **b**, 2 μm; **d,e** 1 μm

Supplementary Fig. 14

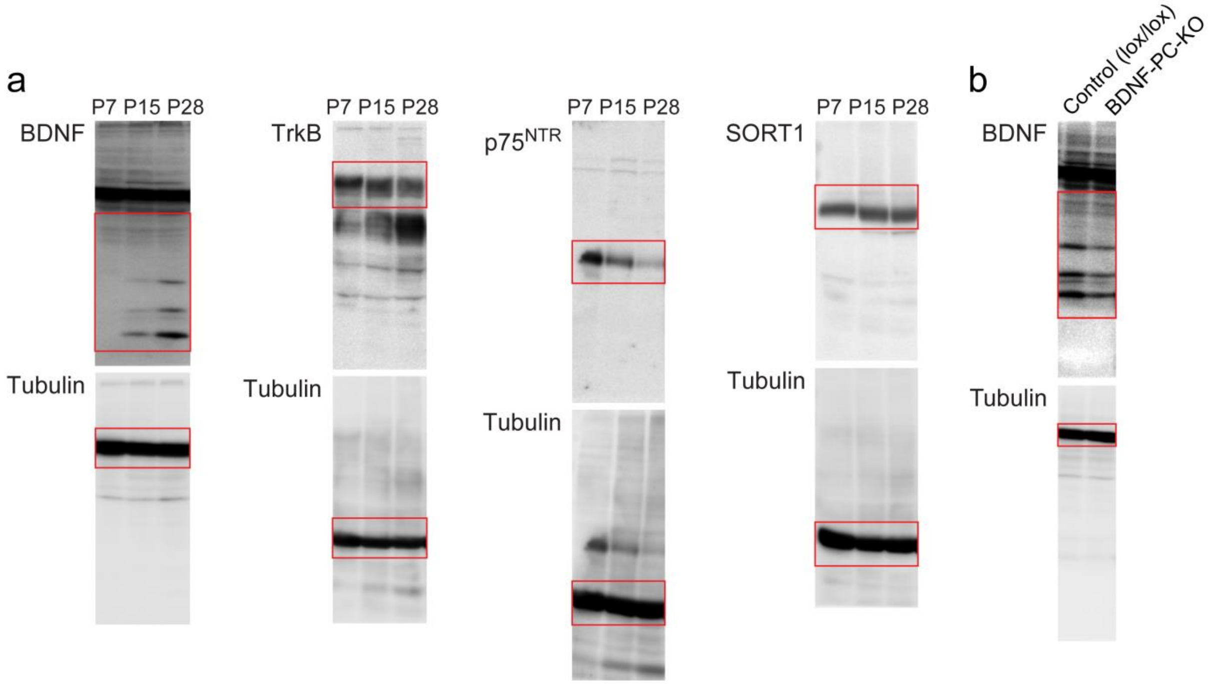

Supplementary Fig. 14. Uncropped images of western blots corresponding to Supplementary Fig. 1 (**a**) and Supplementary Fig. 2 (**b**).

Supplementary tables

Supplementary Table 1

	CF group	Amplitude (nA)	PPR (interval: 50ms)	10-90% rise time (ms)	Decay time constant (ms)	n
Control (P21 - 50)	CF-mono	2.05 ± 0.10	0.79 ± 0.01	0.40 ± 0.02	5.71 ± 0.27	42
	CF-multi-S	1.60 ± 0.22	0.78 ± 0.01	0.39 ± 0.03	5.76 ± 0.39	8
	CF-multi-W	0.41 ± 0.07	0.66 ± 0.03	0.47 ± 0.02	4.81 ± 0.88	5
BDNF-PC-KO (P21 - 50)	CF-mono	2.25 ± 0.13	0.79 ± 0.01	0.46 ± 0.02*	6.13 ± 0.18	43
	CF-multi-S	2.30 ± 0.23	0.75 ± 0.02	0.52 ± 0.03*	5.65 ± 0.26	12
	CF-multi-W	0.63 ± 0.08	0.70 ± 0.05	0.48 ± 0.02	5.05 ± 1.14	6
Control (P10 - 12)	CF-mono	2.48 ± 0.31	0.71 ± 0.02	0.47 ± 0.05	5.98 ± 0.27	10
	CF-multi-S	1.96 ± 0.21	0.70 ± 0.02	0.57 ± 0.03	5.19 ± 0.36	17
	CF-multi-W	0.59 ± 0.14	0.72 ± 0.03	0.59 ± 0.05	4.56 ± 0.44	22
BDNF-PC-KO (P10 - 12)	CF-mono	2.70 ± 0.27	0.71 ± 0.03	0.49 ± 0.01	5.21 ± 0.29	18
	CF-multi-S	2.41 ± 0.27	0.70 ± 0.02	0.55 ± 0.03	5.12 ± 0.32	21
	CF-multi-W	0.59 ± 0.08	0.66 ± 0.02	0.47 ± 0.02	3.77 ± 0.25	25
Control (P13 - 15)	CF-mono	1.86 ± 0.08	0.76 ± 0.01	0.47 ± 0.01	6.23 ± 0.19	33
	CF-multi-S	1.92 ± 0.16	0.73 ± 0.02	0.46 ± 0.02	5.80 ± 0.25	15
	CF-multi-W	0.64 ± 0.09	0.71 ± 0.02	0.47 ± 0.04	4.08 ± 0.39	17
BDNF-PC-KO (P13 - 15)	CF-mono	2.09 ± 0.15	0.75 ± 0.01	0.48 ± 0.02	6.49 ± 0.35	28
	CF-multi-S	1.78 ± 0.13	0.71 ± 0.01	0.53 ± 0.05	5.56 ± 0.28	28
	CF-multi-W	0.54 ± 0.09	0.66 ± 0.02	0.51 ± 0.03	3.78 ± 0.33	26
Control (P16 - 20)	CF-mono	2.08 ± 0.13	0.77 ± 0.01	0.43 ± 0.02	6.33 ± 0.26	27
	CF-multi-S	1.74 ± 0.27	0.69 ± 0.09	0.44 ± 0.02	6.17 ± 0.48	5
	CF-multi-W	0.59 ± 0.19	0.75 ± 0.03	0.42 ± 0.02	4.67 ± 0.48	7
BDNF-PC-KO (P16 - 20)	CF-mono	1.88 ± 0.11	0.76 ± 0.01	0.43 ± 0.02	6.19 ± 0.30	21
	CF-multi-S	1.80 ± 0.15	0.72 ± 0.02	0.44 ± 0.01	6.15 ± 0.44	16
	CF-multi-W	0.47 ± 0.11	0.73 ± 0.02	0.47 ± 0.04	4.31 ± 0.39	15

Supplementary Table 1. Electrophysiological parameters of CF-EPSC in BDNF-PC-KO mice
Amplitudes were measured at holding potential of -10 mV. Rise time was defined as time growing 10%-90% amplitude. PPR (Paired-pulse ratio) was defines as the percent of the second EPSC amplitude relative to the first one. Inter-stimulus interval was 50 ms. The decay time constant was obtained by fitting the EPSC decay with a single exponential. *P*-value was determined by Mann-Whitney *U*-test. All data are expressed as mean ± SEM.

Supplementary Table 2

		Total Amplitude (nA)	Disparity ratio	Disparity index
P21 - 50	Control	2.08 ± 0.10 (n=69)	0.50 ± 0.09 (n=14)	0.58 ± 0.13 (n=14)
	BDNF-PC-KO	2.05 ± 0.10 (n=59)	0.39 ± 0.05 (n=37)	0.70 ± 0.06 (n=37)
P10 - 12	Control	2.80 ± 0.28 (n=25)	0.23 ± 0.04 (n=17)	0.76 ± 0.11 (n=17)
	BDNF-PC-KO	2.58 ± 0.17 (n=34)	0.21 ± 0.04 (n=24)	0.78 ± 0.10 (n=24)
P13 - 15	Control	2.06 ± 0.09 (n=49)	0.23 ± 0.04 (n=27)	0.61 ± 0.07 (n=27)
	BDNF-PC-KO	2.20 ± 0.11 (n=61)	0.26 ± 0.03 (n=29)	0.61 ± 0.06 (n=29)
P16 – 20	Control	2.11 ± 0.12 (n=32)	0.16 ± 0.04 (n=8)	0.80 ± 0.12 (n=8)
	BDNF-PC-KO	2.08 ± 0.11 (n=39)	0.22 ± 0.04 (n=15)	0.73 ± 0.10 (n=15)

Supplementary Table 2. Total amplitudes and disparity parameters of CF-EPSCs in BDNF-PC-KO mice

Amplitudes were measured at holding potential of -10 mV. Disparity parameters were calculated as described in Methods. *P*-value was determined by student's t test. All data are expressed as mean ± SEM.

Supplementary Table 3

	CF group	Amplitude (nA)	PPR (interval: 50ms)	10-90% rise time (ms)	Decay time constant (ms)	n
Control (P21 - 46)	CF-mono	1.79 ± 0.09	0.72 ± 0.04	0.42 ± 0.02	6.67 ± 0.28	35
	CF-multi-S	1.36 ± 0.35	0.77 ± 0.17	0.42 ± 0.10	7.03 ± 1.70	4
	CF-multi-W	0.45 ± 0.17	0.71 ± 0.16	0.46 ± 0.11	4.56 ± 1.33	4
BDNF-PC-KD (P21 - 46)	CF-mono	1.98 ± 0.13	0.74 ± 0.04	0.43 ± 0.02	5.51 ± 0.33*	24
	CF-multi-S	1.75 ± 0.28	0.72 ± 0.09	0.42 ± 0.05	5.70 ± 0.74	8
	CF-multi-W	0.48 ± 0.13	0.72 ± 0.06	0.46 ± 0.05	3.84 ± 0.59	12
Control (P10 - 12)	CF-mono	2.18 ± 0.14	0.72 ± 0.01	0.47 ± 0.02	5.57 ± 0.28	13
	CF-multi-S	1.75 ± 0.16	0.72 ± 0.01	0.53 ± 0.03	5.73 ± 0.28	19
	CF-multi-W	0.60 ± 0.12	0.68 ± 0.02	0.55 ± 0.01	3.47 ± 0.18	21
BDNF-PC-KD (P10 - 12)	CF-mono	2.63 ± 0.35	0.68 ± 0.03	0.52 ± 0.02	5.35 ± 0.44	9
	CF-multi-S	1.75 ± 0.33	0.70 ± 0.02	0.48 ± 0.03	5.63 ± 0.29	8
	CF-multi-W	0.43 ± 0.11	0.68 ± 0.04	0.54 ± 0.05	3.66 ± 0.28	10
Control (P13 - 15)	CF-mono	1.90 ± 0.15	0.76 ± 0.03	0.43 ± 0.02	6.53 ± 0.45	11
	CF-multi-S	1.76 ± 0.22	0.69 ± 0.03	0.46 ± 0.10	5.84 ± 0.51	8
	CF-multi-W	0.82 ± 0.18	0.73 ± 0.03	0.51 ± 0.11	4.97 ± 0.86	7
BDNF-PC-KD (P13 - 15)	CF-mono	2.47 ± 0.46	0.72 ± 0.03	0.44 ± 0.02	6.10 ± 0.33	13
	CF-multi-S	2.03 ± 0.24	0.70 ± 0.03	0.46 ± 0.05	5.45 ± 0.34	9
	CF-multi-W	0.42 ± 0.10	0.72 ± 0.06	0.48 ± 0.05	3.43 ± 0.28	10
Control (P16 – 19)	CF-mono	2.13 ± 0.15	0.78 ± 0.01	0.38 ± 0.02	6.02 ± 0.26	16
	CF-multi-S	1.32 ± 0.08	0.73 ± 0.01	0.40 ± 0.10	7.22 ± 0.36	5
	CF-multi-W	0.51 ± 0.13	0.61 ± 0.07	0.47 ± 0.11	4.35 ± 0.50	5
BDNF-PC-KD (P16 - 19)	CF-mono	2.29 ± 0.17	0.76 ± 0.02	0.37 ± 0.02	5.78 ± 0.33	17
	CF-multi-S	1.76 ± 0.18	0.74 ± 0.03	0.45 ± 0.05	6.17 ± 0.44	11
	CF-multi-W	0.78 ± 0.10	0.63 ± 0.02	0.50 ± 0.05	3.92 ± 0.27	14

Supplementary Table 3. Electrophysiological parameters of CF-EPSC in BDNF-KD PCs

Supplementary Table 4

		Total Amplitude (nA)	Disparity ratio	Disparity index
P21 – 46	Control	1.77 ± 0.07 (n=45)	0.44 ± 0.10 (n=8)	0.61 ± 0.13 (n=8)
	BDNF-PC-KD	1.99 ± 0.11 (n=44)	0.30 ± 0.06 (n=16)	0.84 ± 0.10 (n=16)
P10 – 12	Control	2.42 ± 0.13 (n=39)	0.40 ± 0.04 (n=28)	0.70 ± 0.07 (n=28)
	BDNF-PC-KD	2.31 ± 0.21 (n=29)	0.35 ± 0.06 (n=19)	0.77 ± 0.09 (n=19)
P13 – 15	Control	2.19 ± 0.15 (n=19)	0.34 ± 0.10 (n=13)	0.65 ± 0.14 (n=13)
	BDNF-PC-KD	2.54 ± 0.27 (n=25)	0.23 ± 0.05 (n=13)	0.97 ± 0.10 (n=13)
P16 – 19	Control	2.15 ± 0.13 (n=21)	0.31 ± 0.07 (n=5)	0.77 ± 0.13 (n=5)
	BDNF-PC-KD	2.37 ± 0.13 (n=30)	0.34 ± 0.05 (n=12)	0.57 ± 0.07 (n=12)

Supplementary Table 4. Total amplitudes and disparity parameters of CF-EPSCs in BDNF KD PCs

Supplementary Table 5

	CF group	Amplitude (nA)	PPR (interval: 50ms)	10-90% rise time (ms)	Decay time constant (ms)	n
Control	CF-mono	2.64 ± 0.14	0.77 ± 0.03	0.44 ± 0.02	5.83 ± 0.28	24
	CF-multi-S	2.21 ± 0.29	0.75 ± 0.07	0.39 ± 0.06	5.80 ± 0.59	11
	CF-multi-W	1.42 ± 0.35	0.76 ± 0.12	0.47 ± 0.02	5.25 ± 0.93	6
TrkB-PC-KO	CF-mono	2.76 ± 0.17	0.79 ± 0.02	0.42 ± 0.02	5.62 ± 0.24	35
	CF-multi-S	2.02 ± 0.36	0.76 ± 0.09	0.40 ± 0.05	6.06 ± 0.84	8
	CF-multi-W	1.15 ± 0.35	0.79 ± 0.23	0.46 ± 0.13	4.84 ± 1.48	3
Control	CF-mono	2.48 ± 0.16	0.78 ± 0.03	0.42 ± 0.02	5.26 ± 0.25	26
	CF-multi-S	2.72 ± 0.77	0.73 ± 0.11	0.42 ± 0.07	4.31 ± 0.71	6
	CF-multi-W	1.11 ± 0.38	0.72 ± 0.17	0.41 ± 0.10	4.59 ± 1.25	4
$p75^{NTR}$-PC-KO	CF-mono	2.57 ± 0.21	0.77 ± 0.03	0.44 ± 0.02	5.79 ± 0.33	23
	CF-multi-S	1.96 ± 0.27	0.74 ± 0.08	0.49 ± 0.06	5.87 ± 0.72*	9
	CF-multi-W	1.18 ± 0.18	0.74 ± 0.09	0.45 ± 0.06	4.71 ± 0.76	8

Supplementary Table 5. Electrophysiological parameters of CF-EPSC in TrkB-PC-KO and $p75^{NTR}$-PC-KO mice

Supplementary Table 6

	Total Amplitude (nA)	Disparity ratio	Disparity index
Control	2.54 ± 0.08 (n=49)	0.39 ± 0.10 (n=14)	0.74 ± 0.14 (n=14)
TrkB-PC-KO	2.63 ± 0.12 (n=56)	0.53 ± 0.08 (n=15)	0.52 ± 0.10 (n=15)
Control	2.61 ± 0.16 (n=40)	0.56 ± 0.09 (n=9)	0.48 ± 0.13 (n=9)
$p75^{NTR}$-PC-KO	2.41 ± 0.11 (n=52)	0.50 ± 0.09 (n=13)	0.57 ± 0.13 (n=13)

Supplementary Table 6. Total amplitudes and disparity parameters of CF-EPSCs in TrkB-PC-KO and $p75^{NTR}$-PC-KO mice

Supplementary Table 7

	CF group	Amplitude (nA)	PPR (interval: 50ms)	10-90% rise time (ms)	Decay time constant (ms)	n
Control	CF-mono	2.30 ± 0.14	0.78 ± 0.03	0.42 ± 0.02	5.94 ± 0.23	31
	CF-multi-S	3.19 ± 1.29	0.73 ± 0.17	0.44 ± 0.11	5.65 ± 1.44	4
	CF-multi-W	0.74 ± 0.39	0.77 ± 0.31	0.48 ± 0.20	4.32 ± 1.80	2
TrkB-CF- KD	CF-mono	2.50 ± 0.20	0.76 ± 0.03	0.46 ± 0.02*	5.86 ± 0.26	33
	CF-multi-S	1.86 ± 0.26	0.73 ± 0.07	0.43 ± 0.04	5.71 ± 0.71	11
	CF-multi-W	0.66 ± 0.16	0.74 ± 0.10	0.52 ± 0.08	5.45 ± 0.91	7
Control	CF-mono	1.95 ± 0.15	0.79 ± 0.01	0.41 ± 0.03	7.49 ± 0.33	32
	CF-multi-S	2.05 ± 0.17	0.74 ± 0.02	0.39 ± 0.04	4.77 ± 0.23	48
	CF-multi-W	0.56 ± 0.25	0.77 ± 0.03	0.64 ± 0.17	6.04 ± 0.94	5
$p75^{NTR}$**-CF-**KD	CF-mono	1.73 ± 0.26	0.71 ± 0.02	0.54 ± 0.04	5.88 ± 0.31	4
	CF-multi-S	1.98 ± 0.28	0.74 ± 0.03	0.51 ± 0.05	6.73 ± 0.82	3
	CF-multi-W	0.52 ± 0.16	0.75 ± 0.07	0.27 ± 0.00	3.93 ± 1.12	2

Supplementary Table 7. Electrophysiological parameters of CF-EPSC in PCs associated with TrkB KD CFs and those associated with $p75^{NTR}$ KD CFs

Supplementary Table 8

	Total Amplitude (nA)	Disparity ratio	Disparity index
Control	2.44 ± 0.16 (n=43)	0.37 ± 0.10 (n=8)	0.72 ± 0.14 (n=8)
TrkB-CF-KD	2.25 ± 0.12 (n=59)	0.26 ± 0.04 (n=24)	0.90 ± 0.07 (n=24)
Control	2.00 ± 0.11 (n=46)	0.27 ± 0.05 (n=11)	0.40 ± 0.16 (n=14)
$p75^{NTR}$–CF-KD	2.00 ± 0.11 (n=62)	0.36 ± 0.10 (n=24)	0.70 ± 0.08 (n=24)

Supplementary Table 8. Total amplitudes and disparity parameters of CF-EPSCs in PCs associated with TrkB KD CFs and those associated with $p75^{NTR}$ CFs
